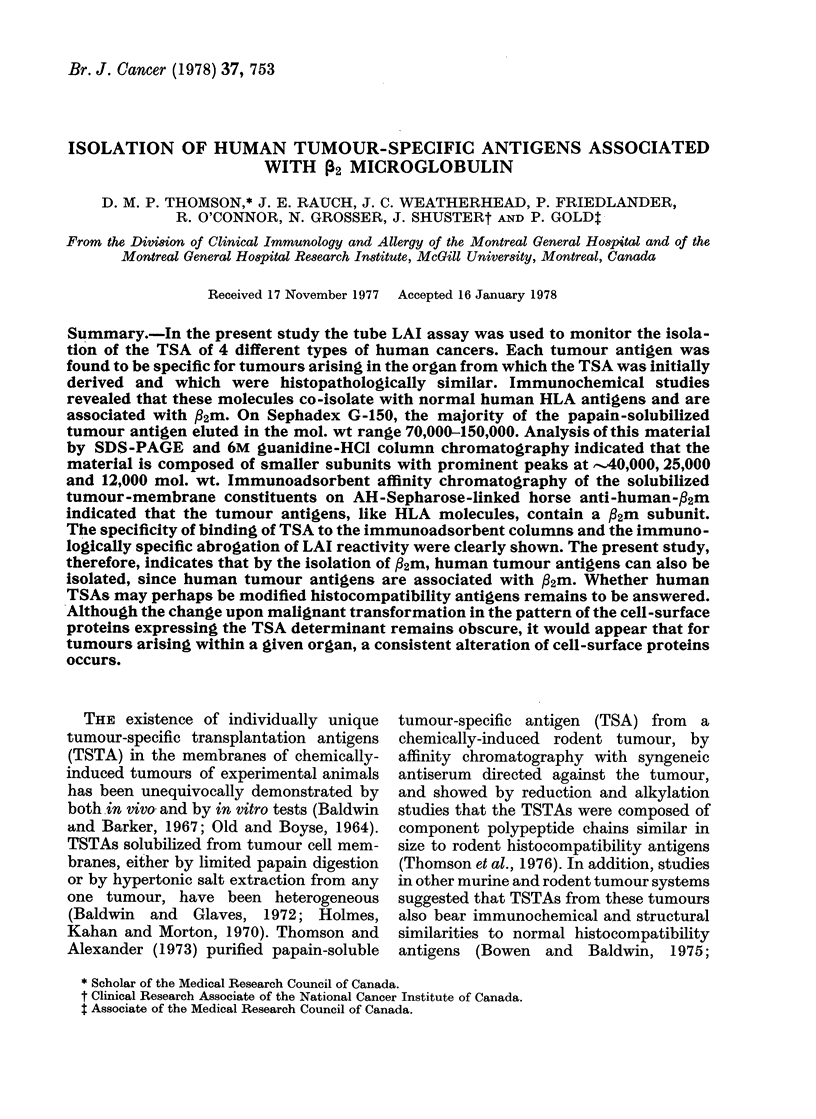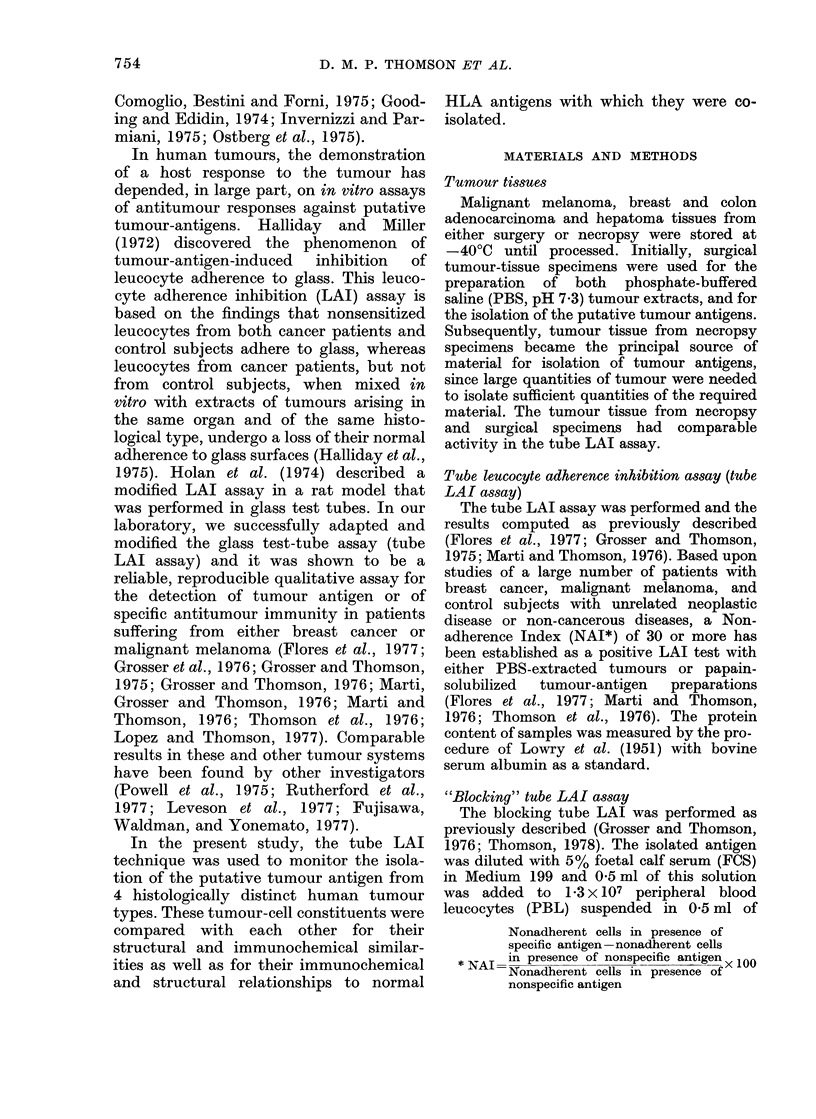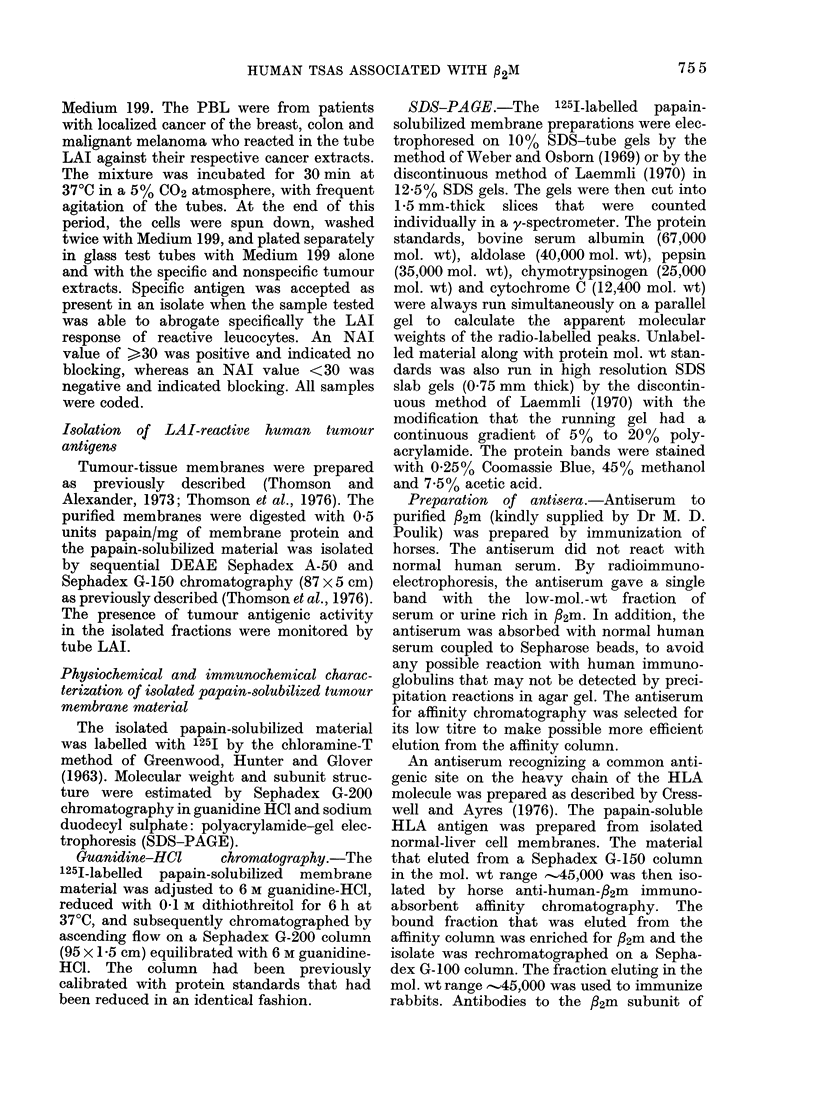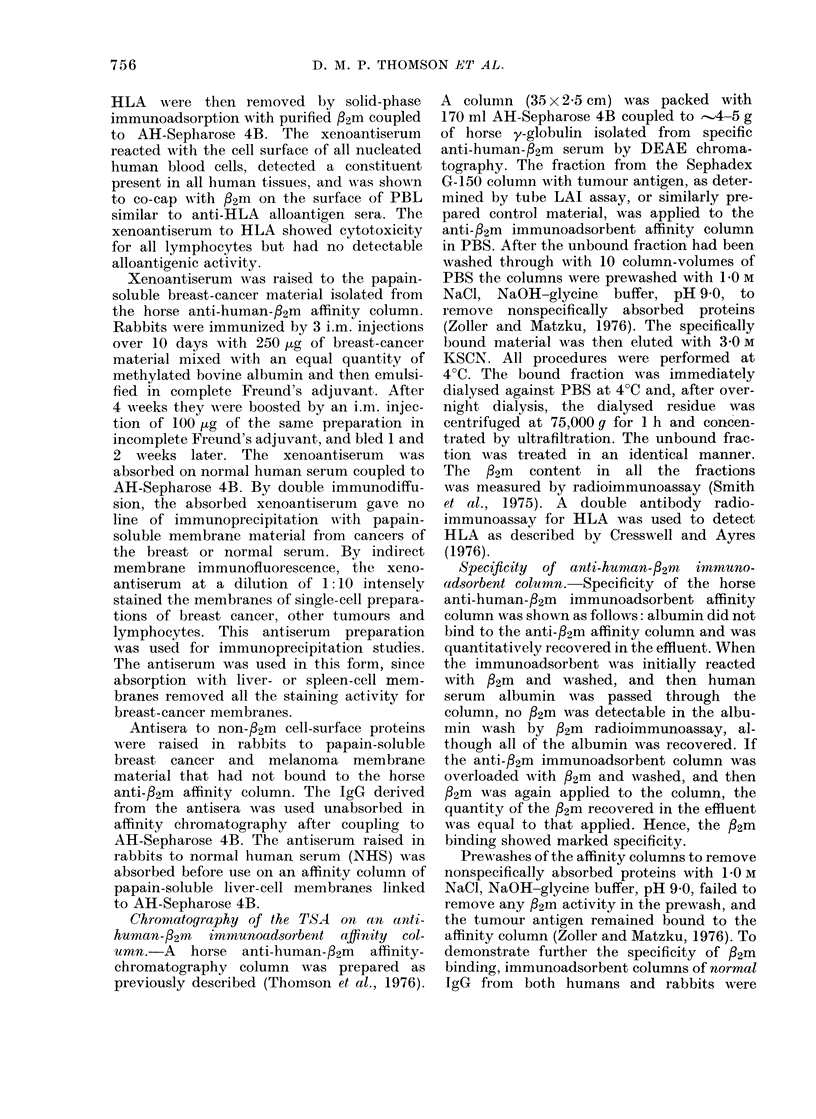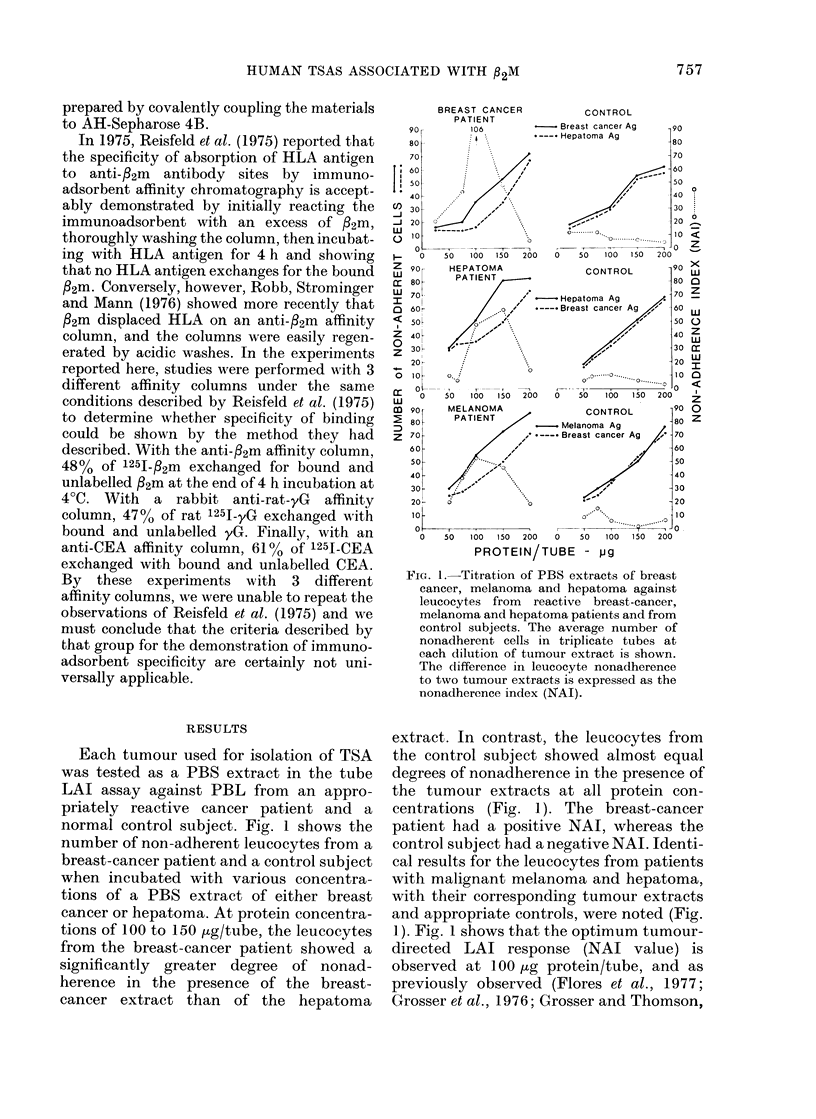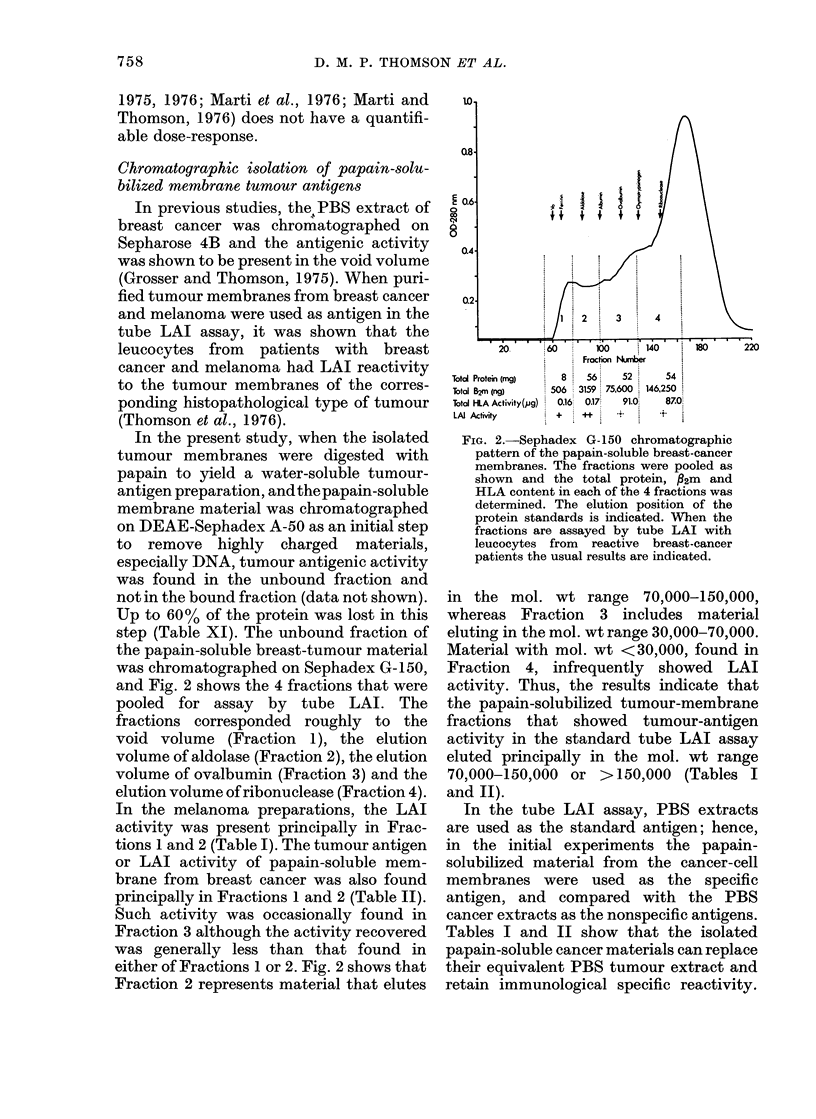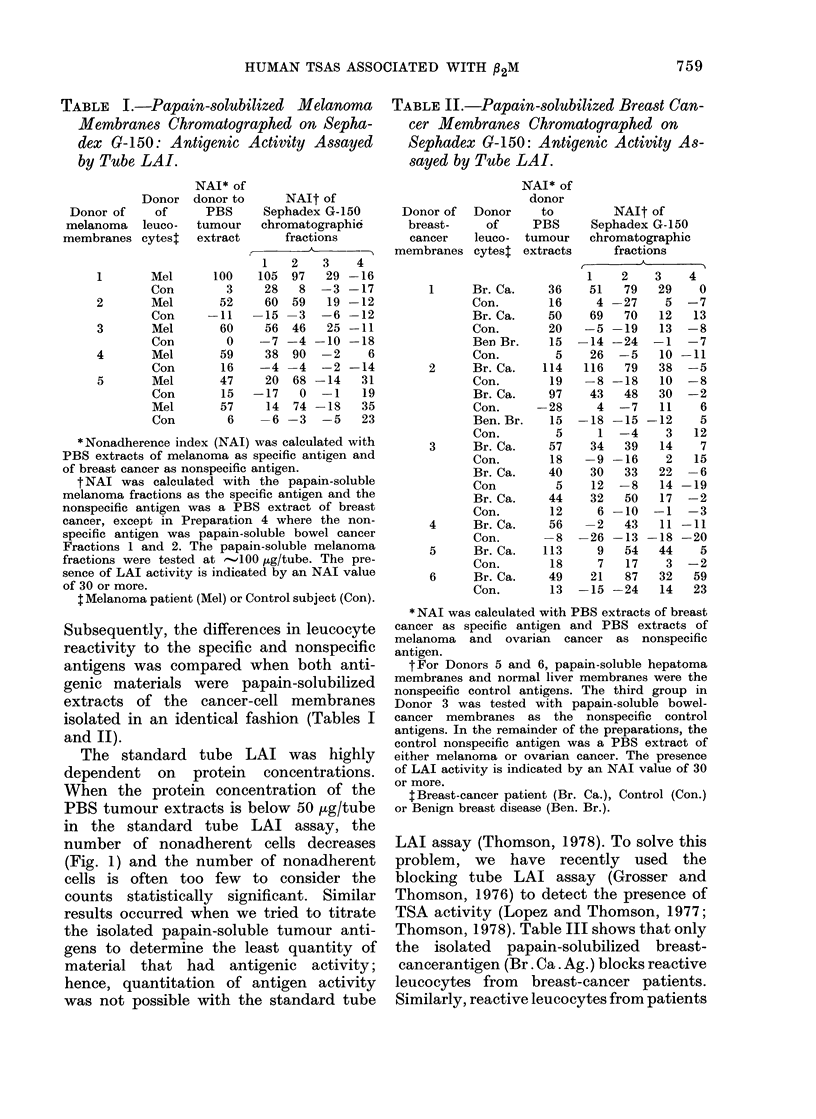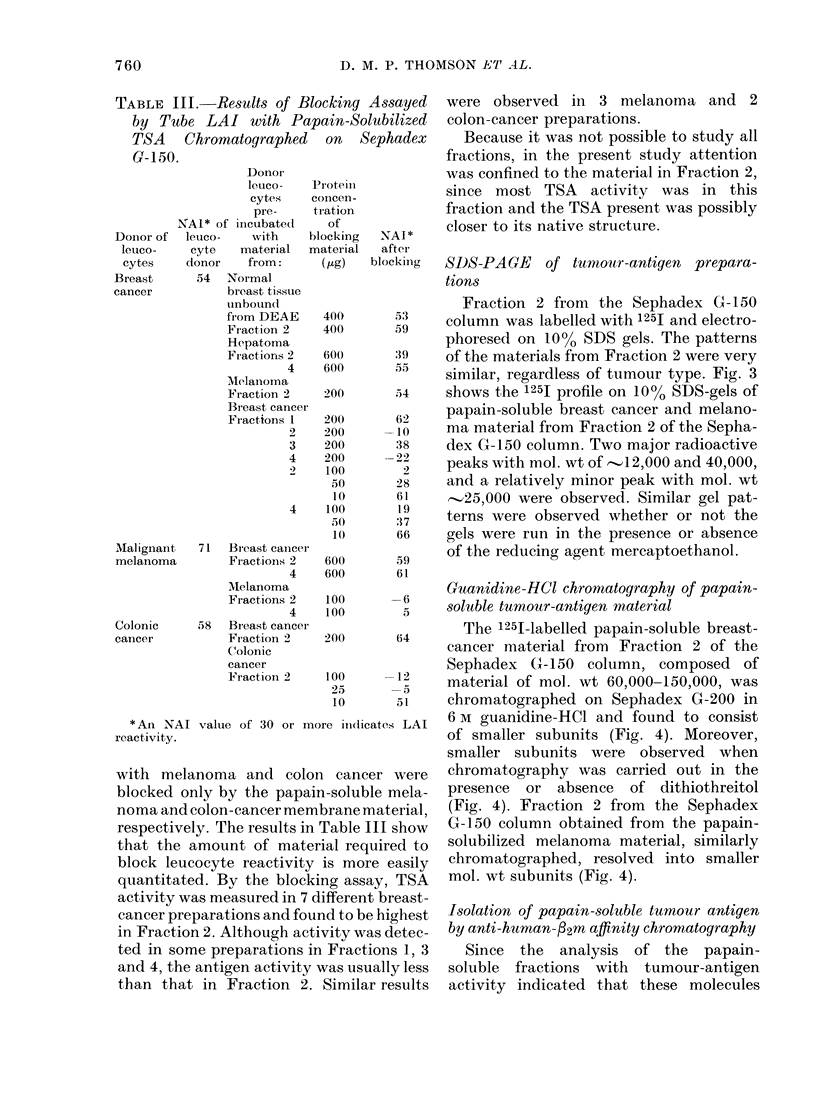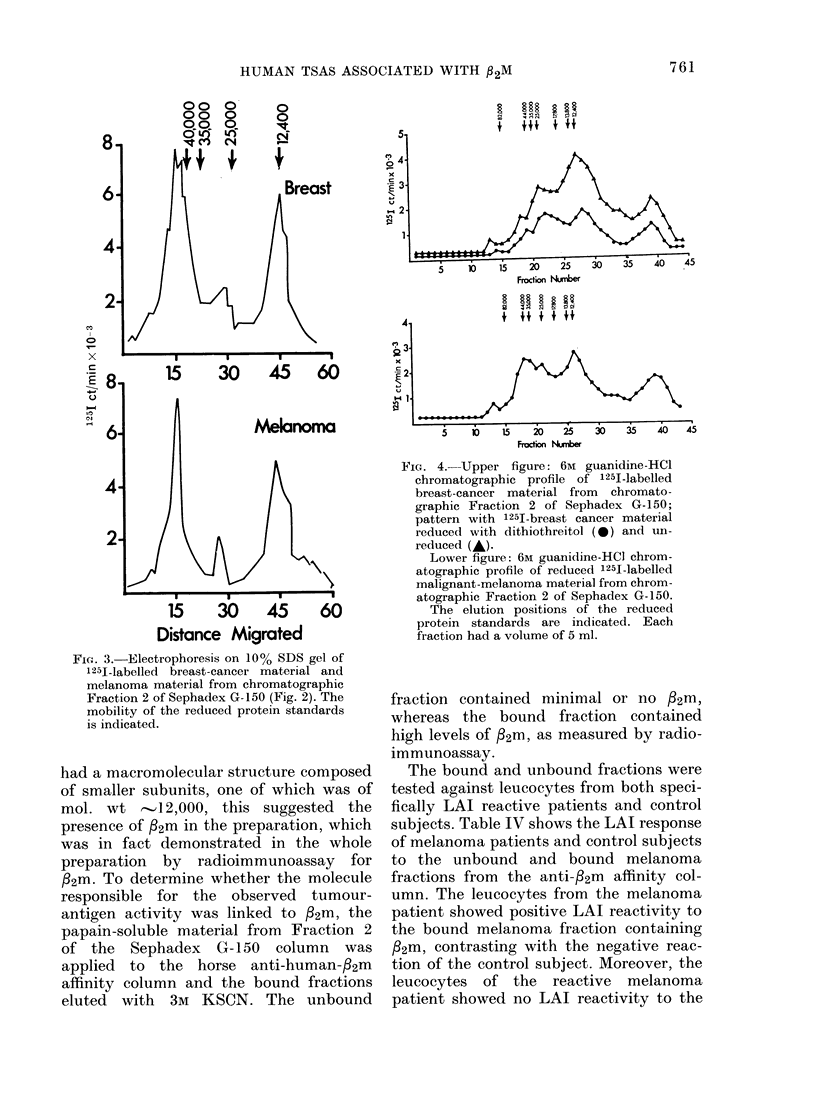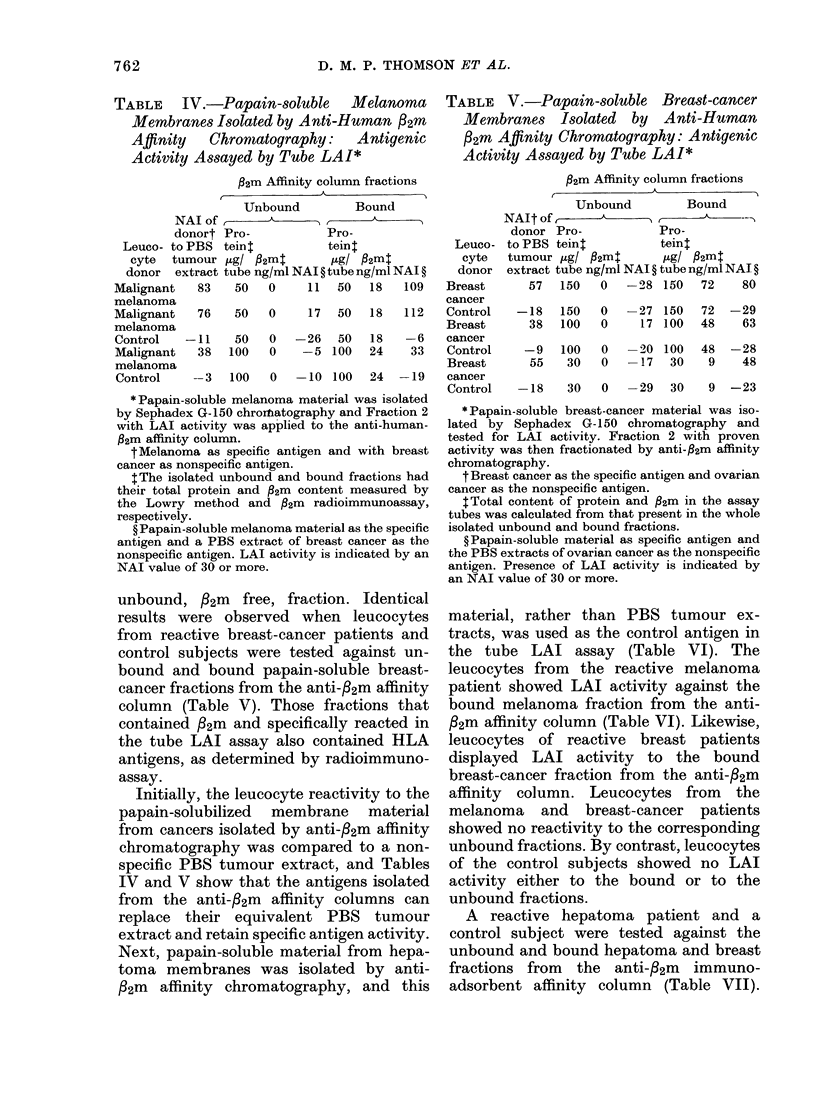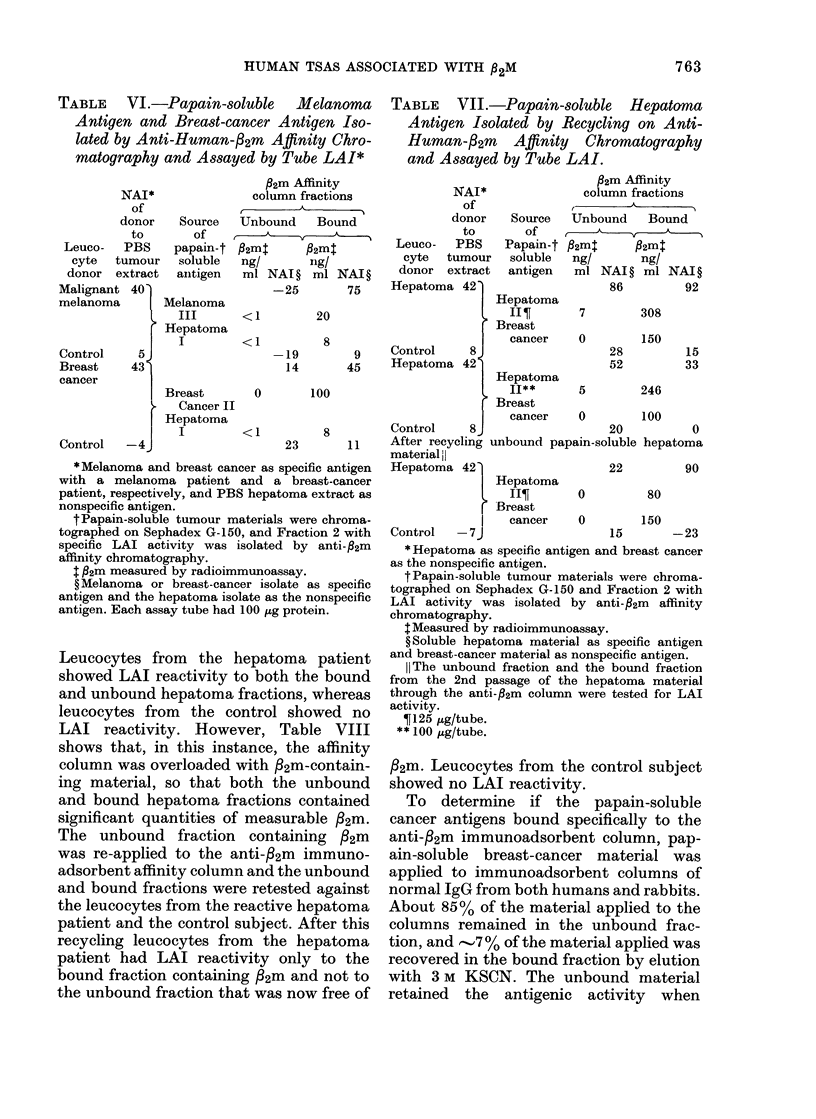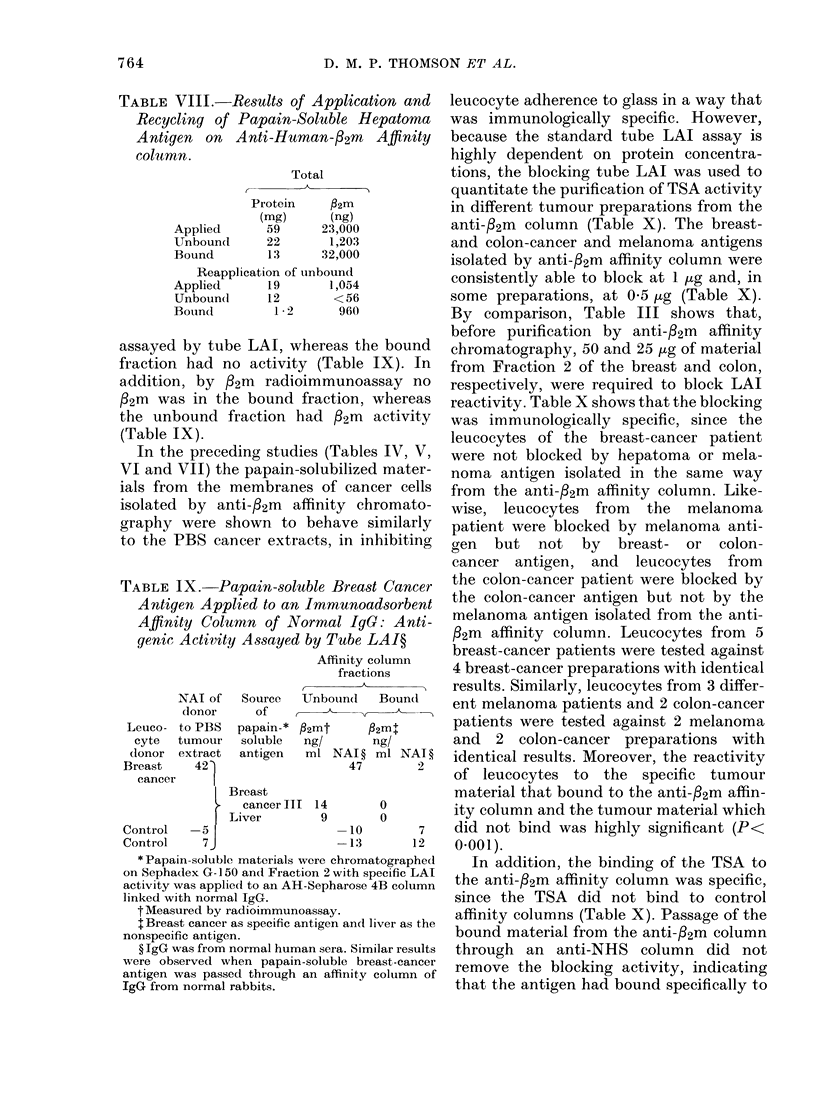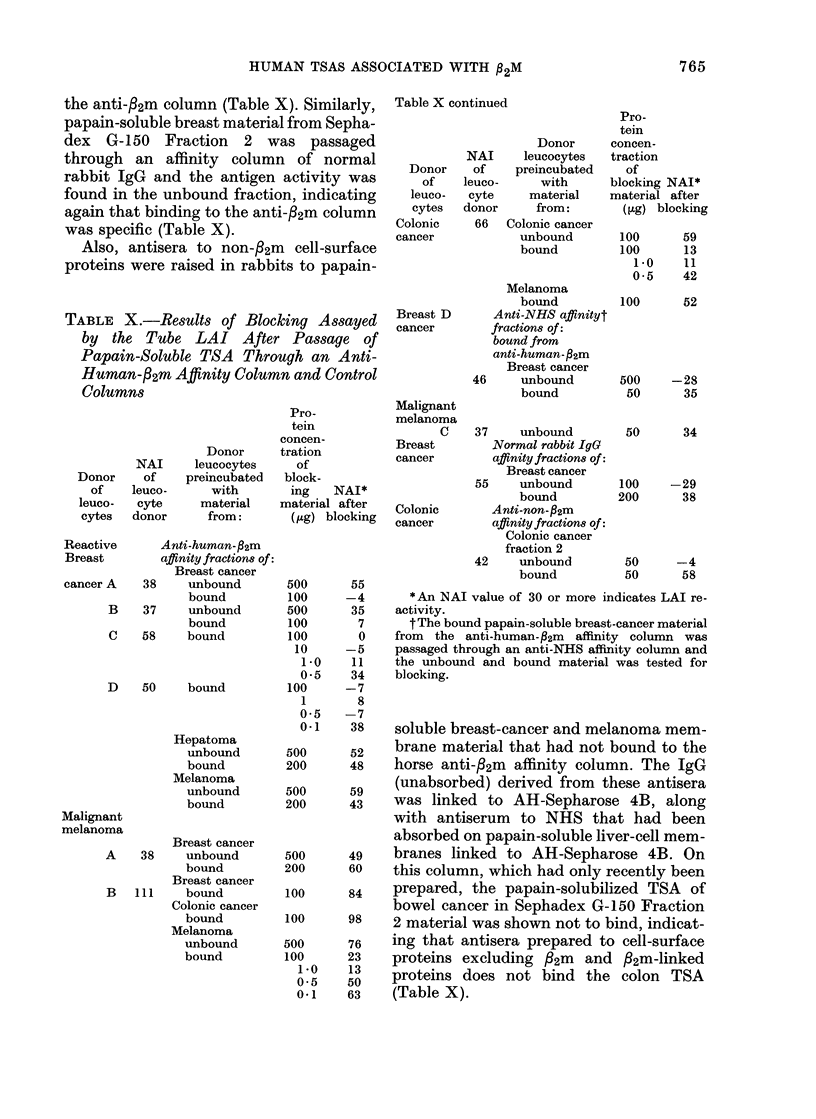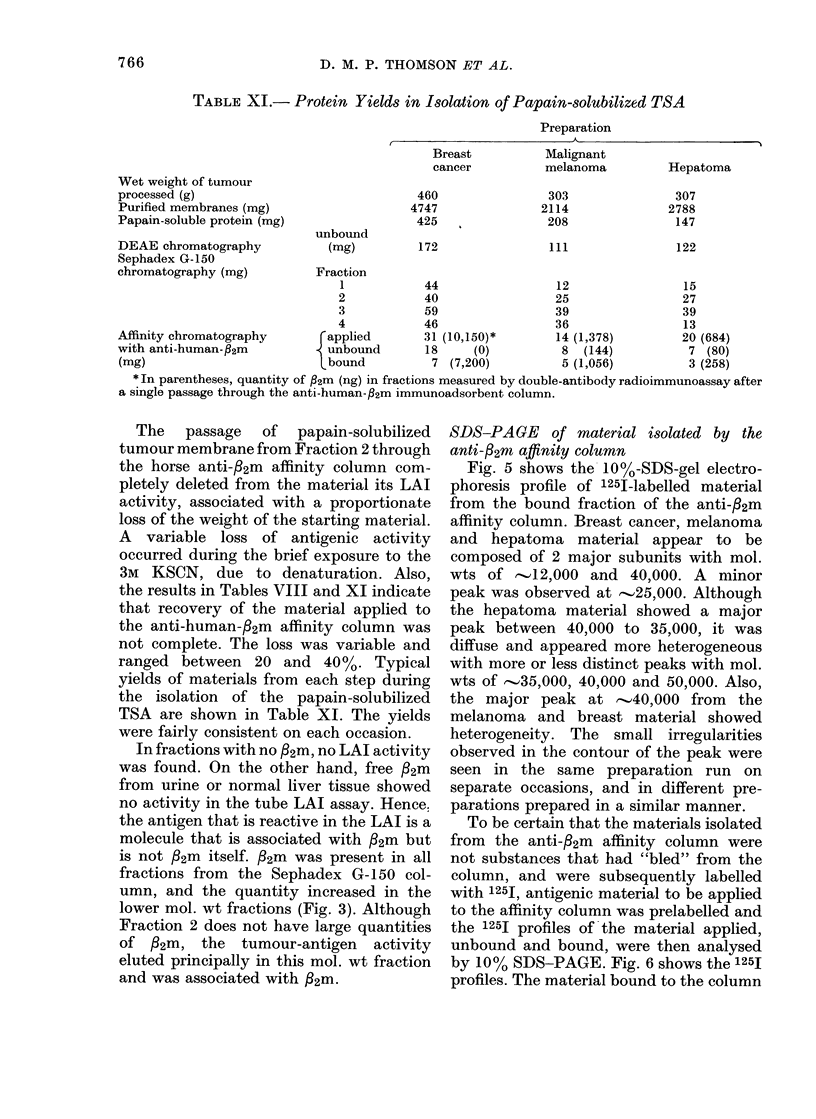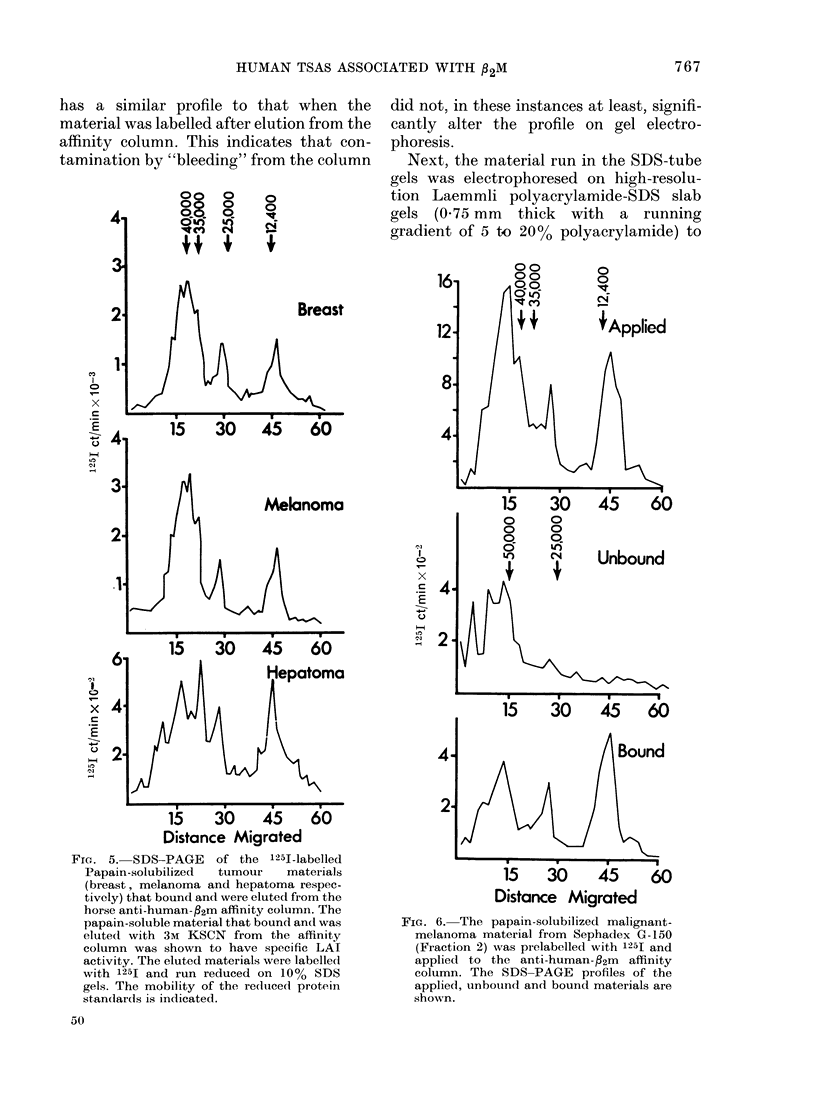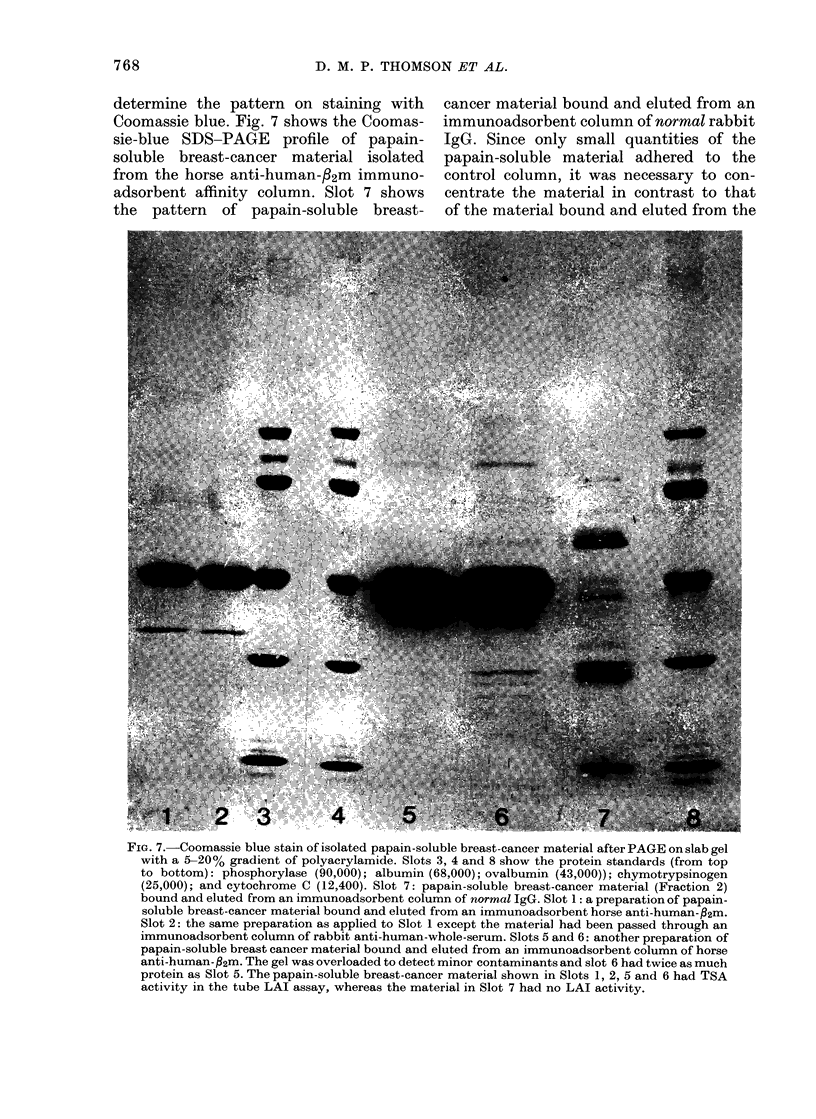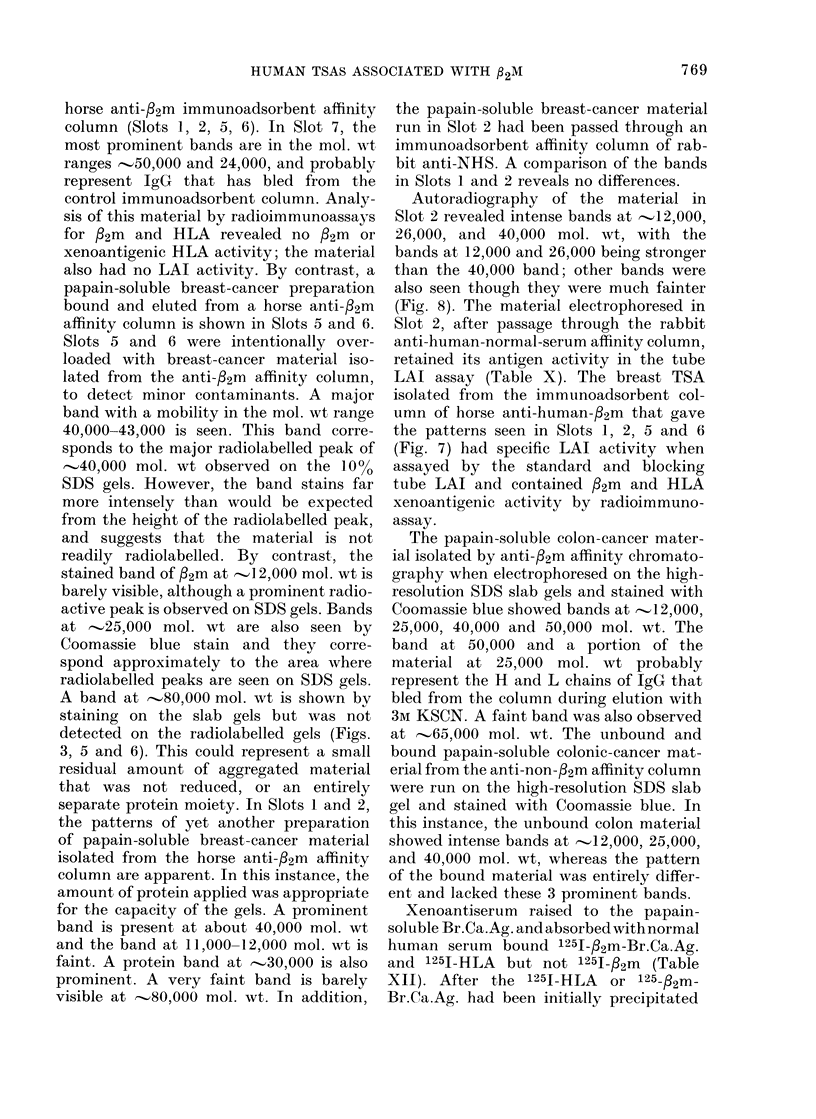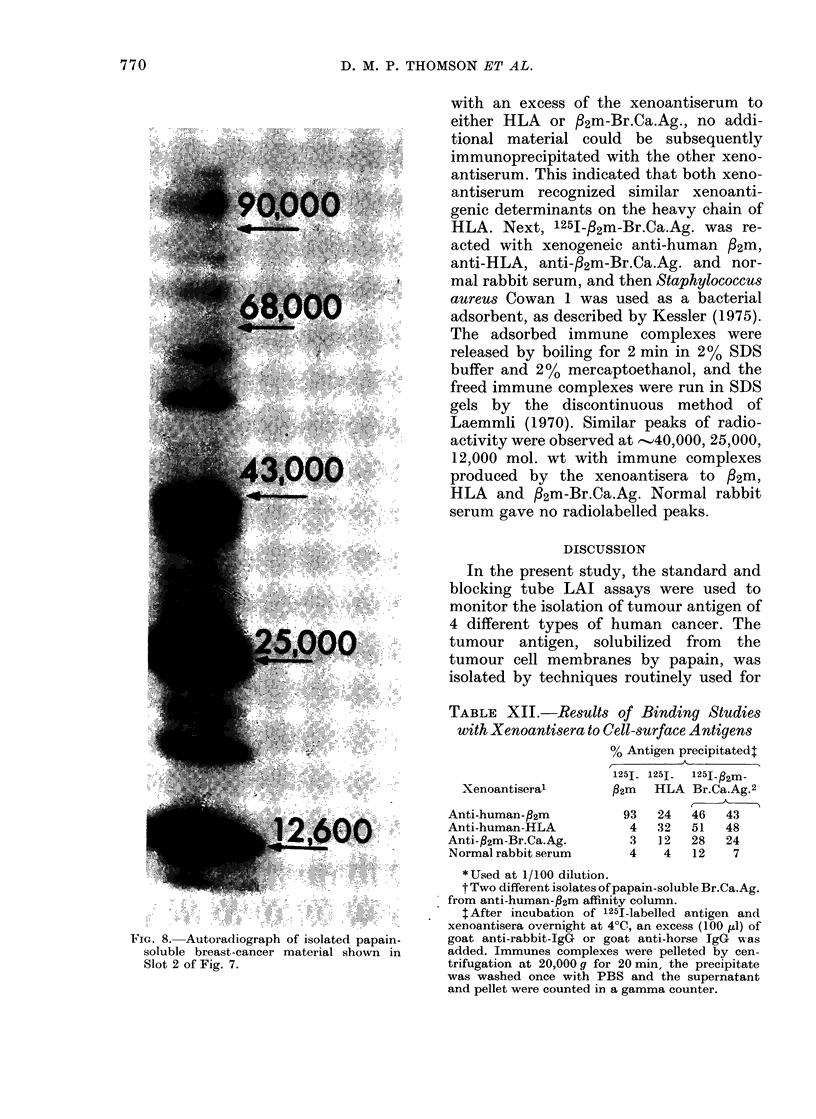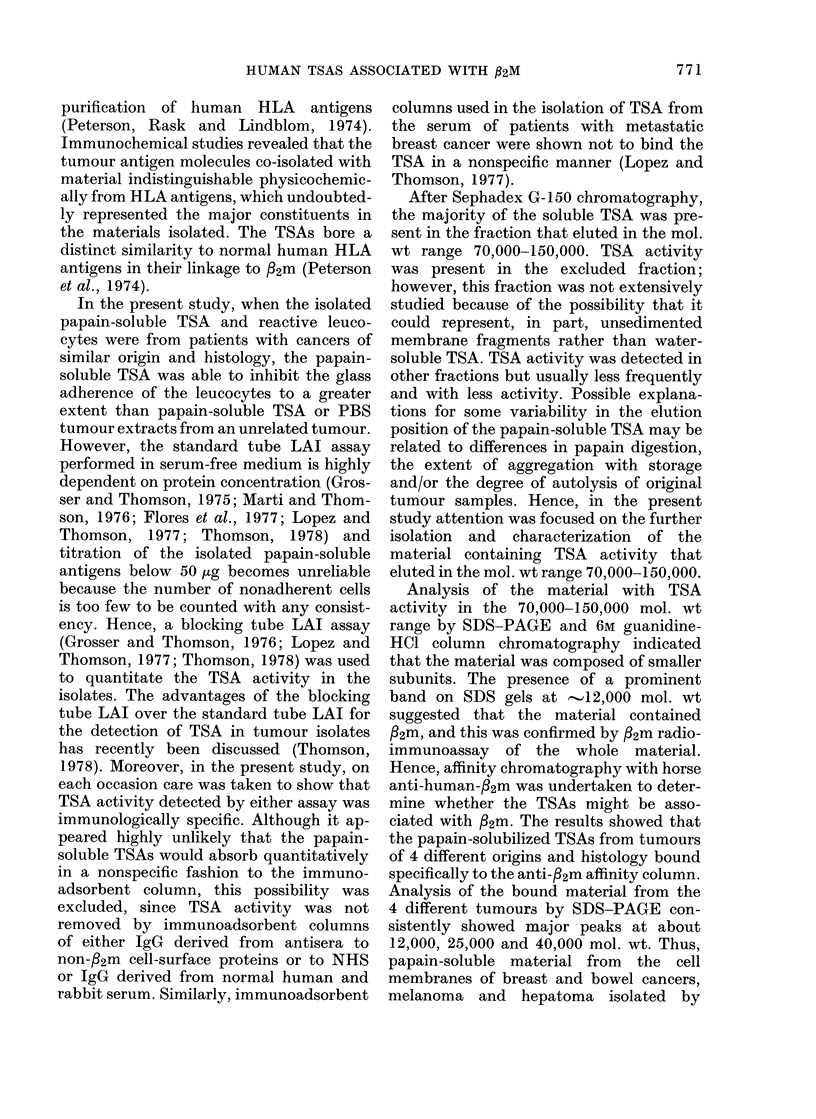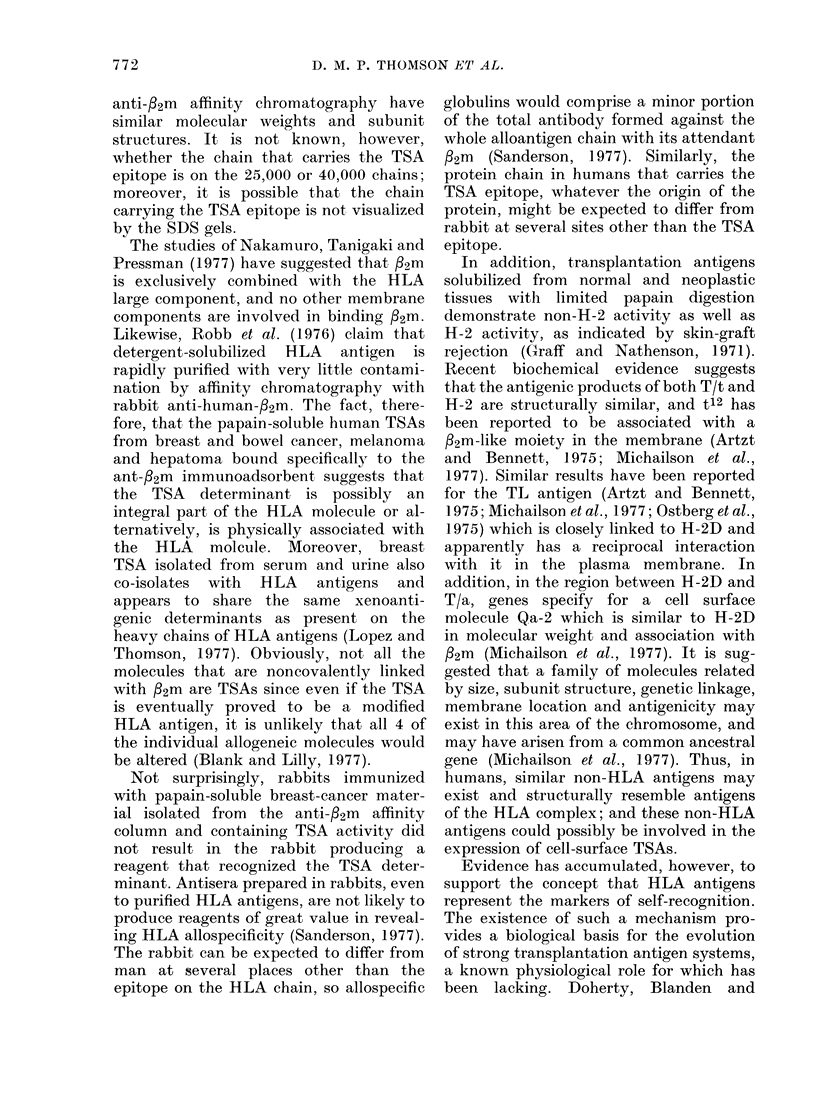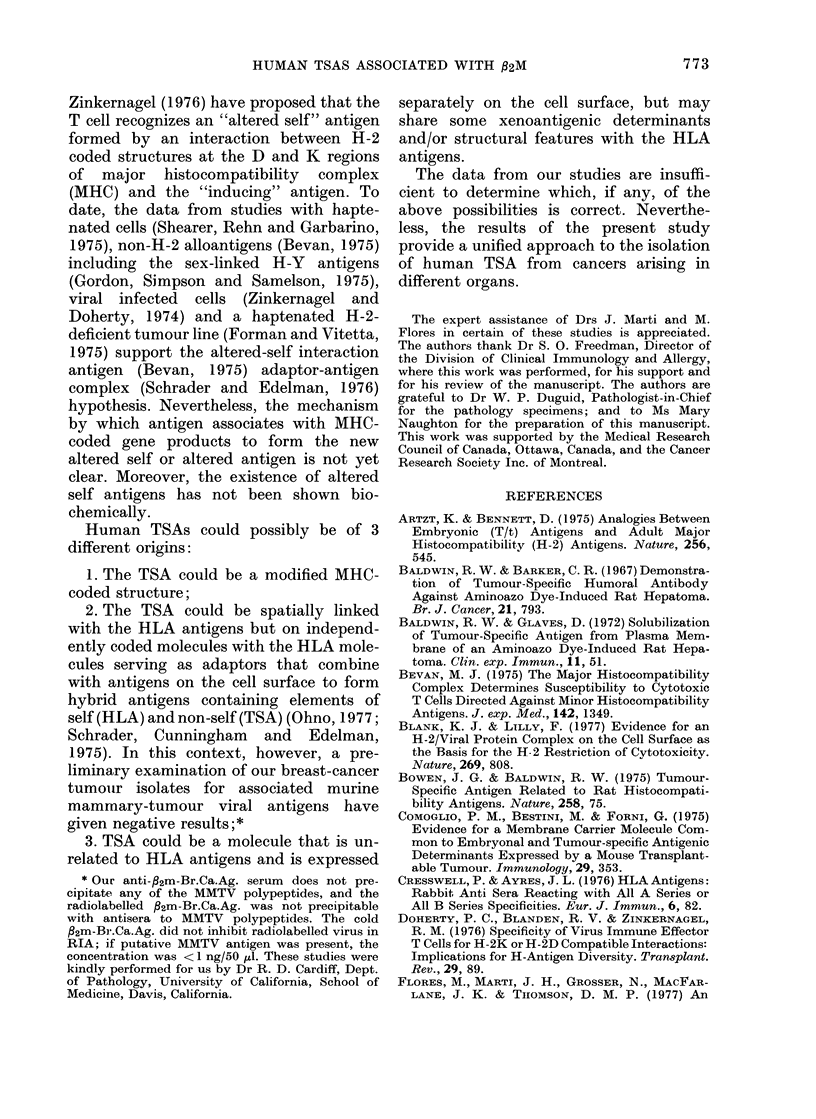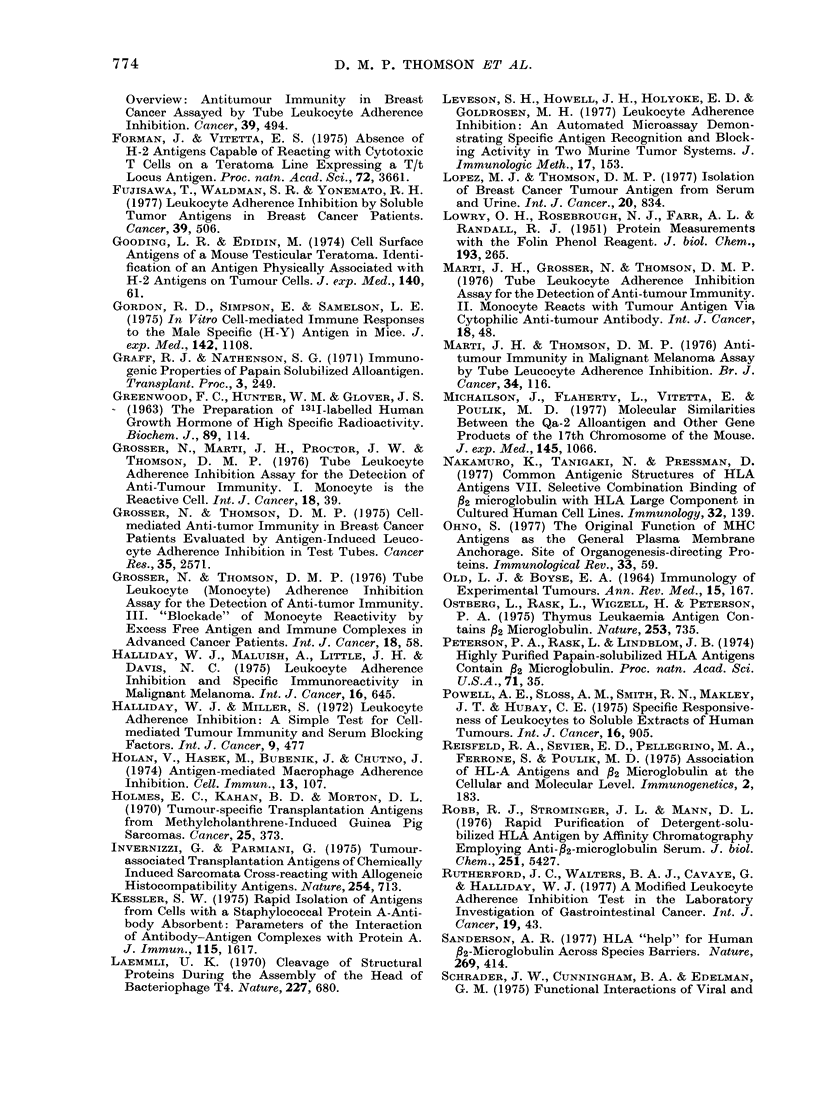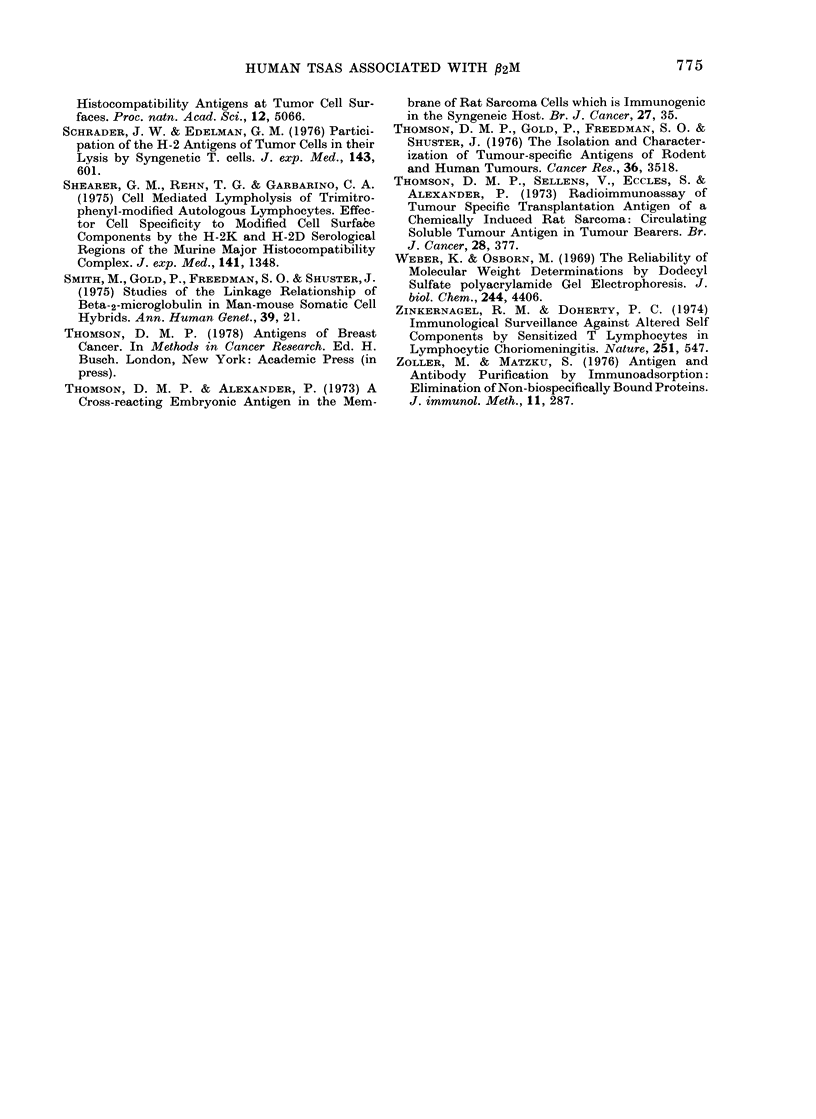# Isolation of human tumour-specific antigens associated with beta2 microglobulin.

**DOI:** 10.1038/bjc.1978.114

**Published:** 1978-05

**Authors:** D. M. Thomson, J. E. Rauch, J. C. Weatherhead, P. Friedlander, R. O'Connor, N. Grosser, J. Shuster, P. Gold

## Abstract

**Images:**


*Br. J. Cancer* (1978) **37**, 753

### ISOLATION OF HUMAN TUMOUR-SPECIFIC ANTIGENS ASSOCIATED WITH $\beta_2$ MICROGLOBULIN

D. M. P. THOMSON,* J. E. RAUCH, J. C. WEATHERHEAD, P. FRIEDLANDER, R. O'CONNOR, N. GROSSER, J. SHUSTER† AND P. GOLD‡

*From the Division of Clinical Immunology and Allergy of the Montreal General Hospital and of the Montreal General Hospital Research Institute, McGill University, Montreal, Canada*

Received 17 November 1977 Accepted 16 January 1978

**Summary.—In the present study the tube LAI assay was used to monitor the isolation of the TSA of 4 different types of human cancers. Each tumour antigen was found to be specific for tumours arising in the organ from which the TSA was initially derived and which were histopathologically similar. Immunochemical studies revealed that these molecules co-isolate with normal human HLA antigens and are associated with $\beta_2$m. On Sephadex G-150, the majority of the papain-solubilized tumour antigen eluted in the mol. wt range 70,000–150,000. Analysis of this material by SDS-PAGE and 6M guanidine-HCl column chromatography indicated that the material is composed of smaller subunits with prominent peaks at ~40,000, 25,000 and 12,000 mol. wt. Immunoadsorbent affinity chromatography of the solubilized tumour-membrane constituents on AH-Sepharose-linked horse anti-human-$\beta_2$m indicated that the tumour antigens, like HLA molecules, contain a $\beta_2$m subunit. The specificity of binding of TSA to the immunoadsorbent columns and the immunologically specific abrogation of LAI reactivity were clearly shown. The present study, therefore, indicates that by the isolation of $\beta_2$m, human tumour antigens can also be isolated, since human tumour antigens are associated with $\beta_2$m. Whether human TSAs may perhaps be modified histocompatibility antigens remains to be answered. Although the change upon malignant transformation in the pattern of the cell-surface proteins expressing the TSA determinant remains obscure, it would appear that for tumours arising within a given organ, a consistent alteration of cell-surface proteins occurs.**

THE existence of individually unique tumour-specific transplantation antigens (TSTA) in the membranes of chemically-induced tumours of experimental animals has been unequivocally demonstrated by both *in vivo* and by *in vitro* tests (Baldwin and Barker, 1967; Old and Boyse, 1964). TSTAs solubilized from tumour cell membranes, either by limited papain digestion or by hypertonic salt extraction from any one tumour, have been heterogeneous (Baldwin and Glaves, 1972; Holmes, Kahan and Morton, 1970). Thomson and Alexander (1973) purified papain-soluble tumour-specific antigen (TSA) from a chemically-induced rodent tumour, by affinity chromatography with syngeneic antiserum directed against the tumour, and showed by reduction and alkylation studies that the TSTAs were composed of component polypeptide chains similar in size to rodent histocompatibility antigens (Thomson *et al.*, 1976). In addition, studies in other murine and rodent tumour systems suggested that TSTAs from these tumours also bear immunochemical and structural similarities to normal histocompatibility antigens (Bowen and Baldwin, 1975;

* Scholar of the Medical Research Council of Canada.
† Clinical Research Associate of the National Cancer Institute of Canada.
‡ Associate of the Medical Research Council of Canada.

Comoglio, Bestini and Forni, 1975; Gooding and Edidin, 1974; Invernizzi and Parmiani, 1975; Ostberg *et al.*, 1975).

In human tumours, the demonstration of a host response to the tumour has depended, in large part, on *in vitro* assays of antitumour responses against putative tumour-antigens. Halliday and Miller (1972) discovered the phenomenon of tumour-antigen-induced inhibition of leucocyte adherence to glass. This leucocyte adherence inhibition (LAI) assay is based on the findings that nonsensitized leucocytes from both cancer patients and control subjects adhere to glass, whereas leucocytes from cancer patients, but not from control subjects, when mixed *in vitro* with extracts of tumours arising in the same organ and of the same histological type, undergo a loss of their normal adherence to glass surfaces (Halliday *et al.*, 1975). Holan *et al.* (1974) described a modified LAI assay in a rat model that was performed in glass test tubes. In our laboratory, we successfully adapted and modified the glass test-tube assay (tube LAI assay) and it was shown to be a reliable, reproducible qualitative assay for the detection of tumour antigen or of specific antitumour immunity in patients suffering from either breast cancer or malignant melanoma (Flores *et al.*, 1977; Grosser *et al.*, 1976; Grosser and Thomson, 1975; Grosser and Thomson, 1976; Marti, Grosser and Thomson, 1976; Marti and Thomson, 1976; Thomson *et al.*, 1976; Lopez and Thomson, 1977). Comparable results in these and other tumour systems have been found by other investigators (Powell *et al.*, 1975; Rutherford *et al.*, 1977; Leveson *et al.*, 1977; Fujisawa, Waldman, and Yonemato, 1977).

In the present study, the tube LAI technique was used to monitor the isolation of the putative tumour antigen from 4 histologically distinct human tumour types. These tumour-cell constituents were compared with each other for their structural and immunochemical similarities as well as for their immunochemical and structural relationships to normal HLA antigens with which they were co-isolated.

### MATERIALS AND METHODS

### *Tumour tissues*

Malignant melanoma, breast and colon adenocarcinoma and hepatoma tissues from either surgery or necropsy were stored at −40°C until processed. Initially, surgical tumour-tissue specimens were used for the preparation of both phosphate-buffered saline (PBS, pH 7·3) tumour extracts, and for the isolation of the putative tumour antigens. Subsequently, tumour tissue from necropsy specimens became the principal source of material for isolation of tumour antigens, since large quantities of tumour were needed to isolate sufficient quantities of the required material. The tumour tissue from necropsy and surgical specimens had comparable activity in the tube LAI assay.

### *Tube leucocyte adherence inhibition assay (tube LAI assay)*

The tube LAI assay was performed and the results computed as previously described (Flores *et al.*, 1977; Grosser and Thomson, 1975; Marti and Thomson, 1976). Based upon studies of a large number of patients with breast cancer, malignant melanoma, and control subjects with unrelated neoplastic disease or non-cancerous diseases, a Non-adherence Index (NAI*) of 30 or more has been established as a positive LAI test with either PBS-extracted tumours or papain-solubilized tumour-antigen preparations (Flores *et al.*, 1977; Marti and Thomson, 1976; Thomson *et al.*, 1976). The protein content of samples was measured by the procedure of Lowry *et al.* (1951) with bovine serum albumin as a standard.

### *"Blocking" tube LAI assay*

The blocking tube LAI was performed as previously described (Grosser and Thomson, 1976; Thomson, 1978). The isolated antigen was diluted with 5% foetal calf serum (FCS) in Medium 199 and 0·5 ml of this solution was added to $1{\cdot}3 \times 10^7$ peripheral blood leucocytes (PBL) suspended in 0·5 ml of

$$* \text{NAI} = \frac{\text{Nonadherent cells in presence of specific antigen} - \text{nonadherent cells in presence of nonspecific antigen}}{\text{Nonadherent cells in presence of nonspecific antigen}} \times 100$$

Medium 199. The PBL were from patients with localized cancer of the breast, colon and malignant melanoma who reacted in the tube LAI against their respective cancer extracts. The mixture was incubated for 30 min at 37°C in a 5% $CO_2$ atmosphere, with frequent agitation of the tubes. At the end of this period, the cells were spun down, washed twice with Medium 199, and plated separately in glass test tubes with Medium 199 alone and with the specific and nonspecific tumour extracts. Specific antigen was accepted as present in an isolate when the sample tested was able to abrogate specifically the LAI response of reactive leucocytes. An NAI value of $\geqslant 30$ was positive and indicated no blocking, whereas an NAI value $<30$ was negative and indicated blocking. All samples were coded.

*Isolation of LAI-reactive human tumour antigens*

Tumour-tissue membranes were prepared as previously described (Thomson and Alexander, 1973; Thomson *et al.*, 1976). The purified membranes were digested with 0·5 units papain/mg of membrane protein and the papain-solubilized material was isolated by sequential DEAE Sephadex A-50 and Sephadex G-150 chromatography (87 × 5 cm) as previously described (Thomson *et al.*, 1976). The presence of tumour antigenic activity in the isolated fractions were monitored by tube LAI.

*Physiochemical and immunochemical characterization of isolated papain-solubilized tumour membrane material*

The isolated papain-solubilized material was labelled with $^{125}$I by the chloramine-T method of Greenwood, Hunter and Glover (1963). Molecular weight and subunit structure were estimated by Sephadex G-200 chromatography in guanidine HCl and sodium duodecyl sulphate: polyacrylamide–gel electrophoresis (SDS–PAGE).

*Guanidine–HCl chromatography.*—The $^{125}$I-labelled papain-solubilized membrane material was adjusted to 6 M guanidine-HCl, reduced with 0·1 M dithiothreitol for 6 h at 37°C, and subsequently chromatographed by ascending flow on a Sephadex G-200 column (95 × 1·5 cm) equilibrated with 6 M guanidine-HCl. The column had been previously calibrated with protein standards that had been reduced in an identical fashion.

*SDS–PAGE.*—The $^{125}$I-labelled papain-solubilized membrane preparations were electrophoresed on 10% SDS–tube gels by the method of Weber and Osborn (1969) or by the discontinuous method of Laemmli (1970) in 12·5% SDS gels. The gels were then cut into 1·5 mm-thick slices that were counted individually in a $\gamma$-spectrometer. The protein standards, bovine serum albumin (67,000 mol. wt), aldolase (40,000 mol. wt), pepsin (35,000 mol. wt), chymotrypsinogen (25,000 mol. wt) and cytochrome C (12,400 mol. wt) were always run simultaneously on a parallel gel to calculate the apparent molecular weights of the radio-labelled peaks. Unlabelled material along with protein mol. wt standards was also run in high resolution SDS slab gels (0·75 mm thick) by the discontinuous method of Laemmli (1970) with the modification that the running gel had a continuous gradient of 5% to 20% polyacrylamide. The protein bands were stained with 0·25% Coomassie Blue, 45% methanol and 7·5% acetic acid.

*Preparation of antisera.*—Antiserum to purified $\beta_2$m (kindly supplied by Dr M. D. Poulik) was prepared by immunization of horses. The antiserum did not react with normal human serum. By radioimmunoelectrophoresis, the antiserum gave a single band with the low-mol.-wt fraction of serum or urine rich in $\beta_2$m. In addition, the antiserum was absorbed with normal human serum coupled to Sepharose beads, to avoid any possible reaction with human immunoglobulins that may not be detected by precipitation reactions in agar gel. The antiserum for affinity chromatography was selected for its low titre to make possible more efficient elution from the affinity column.

An antiserum recognizing a common antigenic site on the heavy chain of the HLA molecule was prepared as described by Cresswell and Ayres (1976). The papain-soluble HLA antigen was prepared from isolated normal-liver cell membranes. The material that eluted from a Sephadex G-150 column in the mol. wt range ~45,000 was then isolated by horse anti-human-$\beta_2$m immunoabsorbent affinity chromatography. The bound fraction that was eluted from the affinity column was enriched for $\beta_2$m and the isolate was rechromatographed on a Sephadex G-100 column. The fraction eluting in the mol. wt range ~45,000 was used to immunize rabbits. Antibodies to the $\beta_2$m subunit of

HLA were then removed by solid-phase immunoadsorption with purified $\beta_2$m coupled to AH-Sepharose 4B. The xenoantiserum reacted with the cell surface of all nucleated human blood cells, detected a constituent present in all human tissues, and was shown to co-cap with $\beta_2$m on the surface of PBL similar to anti-HLA alloantigen sera. The xenoantiserum to HLA showed cytotoxicity for all lymphocytes but had no detectable alloantigenic activity.

Xenoantiserum was raised to the papain-soluble breast-cancer material isolated from the horse anti-human-$\beta_2$m affinity column. Rabbits were immunized by 3 i.m. injections over 10 days with 250 $\mu$g of breast-cancer material mixed with an equal quantity of methylated bovine albumin and then emulsified in complete Freund's adjuvant. After 4 weeks they were boosted by an i.m. injection of 100 $\mu$g of the same preparation in incomplete Freund's adjuvant, and bled 1 and 2 weeks later. The xenoantiserum was absorbed on normal human serum coupled to AH-Sepharose 4B. By double immunodiffusion, the absorbed xenoantiserum gave no line of immunoprecipitation with papain-soluble membrane material from cancers of the breast or normal serum. By indirect membrane immunofluorescence, the xeno-antiserum at a dilution of 1:10 intensely stained the membranes of single-cell preparations of breast cancer, other tumours and lymphocytes. This antiserum preparation was used for immunoprecipitation studies. The antiserum was used in this form, since absorption with liver- or spleen-cell membranes removed all the staining activity for breast-cancer membranes.

Antisera to non-$\beta_2$m cell-surface proteins were raised in rabbits to papain-soluble breast cancer and melanoma membrane material that had not bound to the horse anti-$\beta_2$m affinity column. The IgG derived from the antisera was used unabsorbed in affinity chromatography after coupling to AH-Sepharose 4B. The antiserum raised in rabbits to normal human serum (NHS) was absorbed before use on an affinity column of papain-soluble liver-cell membranes linked to AH-Sepharose 4B.

*Chromatography of the TSA on an anti-human-$\beta_2$m immunoadsorbent affinity column.*—A horse anti-human-$\beta_2$m affinity-chromatography column was prepared as previously described (Thomson *et al.*, 1976). A column (35 × 2·5 cm) was packed with 170 ml AH-Sepharose 4B coupled to ~4–5 g of horse $\gamma$-globulin isolated from specific anti-human-$\beta_2$m serum by DEAE chromatography. The fraction from the Sephadex G-150 column with tumour antigen, as determined by tube LAI assay, or similarly prepared control material, was applied to the anti-$\beta_2$m immunoadsorbent affinity column in PBS. After the unbound fraction had been washed through with 10 column-volumes of PBS the columns were prewashed with 1·0 M NaCl, NaOH–glycine buffer, pH 9·0, to remove nonspecifically absorbed proteins (Zoller and Matzku, 1976). The specifically bound material was then eluted with 3·0 M KSCN. All procedures were performed at 4°C. The bound fraction was immediately dialysed against PBS at 4°C and, after overnight dialysis, the dialysed residue was centrifuged at 75,000 *g* for 1 h and concentrated by ultrafiltration. The unbound fraction was treated in an identical manner. The $\beta_2$m content in all the fractions was measured by radioimmunoassay (Smith *et al.*, 1975). A double antibody radioimmunoassay for HLA was used to detect HLA as described by Cresswell and Ayres (1976).

*Specificity of anti-human-$\beta_2$m immunoadsorbent column.*—Specificity of the horse anti-human-$\beta_2$m immunoadsorbent affinity column was shown as follows: albumin did not bind to the anti-$\beta_2$m affinity column and was quantitatively recovered in the effluent. When the immunoadsorbent was initially reacted with $\beta_2$m and washed, and then human serum albumin was passed through the column, no $\beta_2$m was detectable in the albumin wash by $\beta_2$m radioimmunoassay, although all of the albumin was recovered. If the anti-$\beta_2$m immunoadsorbent column was overloaded with $\beta_2$m and washed, and then $\beta_2$m was again applied to the column, the quantity of the $\beta_2$m recovered in the effluent was equal to that applied. Hence, the $\beta_2$m binding showed marked specificity.

Prewashes of the affinity columns to remove nonspecifically absorbed proteins with 1·0 M NaCl, NaOH–glycine buffer, pH 9·0, failed to remove any $\beta_2$m activity in the prewash, and the tumour antigen remained bound to the affinity column (Zoller and Matzku, 1976). To demonstrate further the specificity of $\beta_2$m binding, immunoadsorbent columns of *normal* IgG from both humans and rabbits were prepared by covalently coupling the materials to AH-Sepharose 4B.

In 1975, Reisfeld *et al.* (1975) reported that the specificity of absorption of HLA antigen to anti-$\beta_2$m antibody sites by immunoadsorbent affinity chromatography is acceptably demonstrated by initially reacting the immunoadsorbent with an excess of $\beta_2$m, thoroughly washing the column, then incubating with HLA antigen for 4 h and showing that no HLA antigen exchanges for the bound $\beta_2$m. Conversely, however, Robb, Strominger and Mann (1976) showed more recently that $\beta_2$m displaced HLA on an anti-$\beta_2$m affinity column, and the columns were easily regenerated by acidic washes. In the experiments reported here, studies were performed with 3 different affinity columns under the same conditions described by Reisfeld *et al.* (1975) to determine whether specificity of binding could be shown by the method they had described. With the anti-$\beta_2$m affinity column, 48% of $^{125}$I-$\beta_2$m exchanged for bound and unlabelled $\beta_2$m at the end of 4 h incubation at 4°C. With a rabbit anti-rat-$\gamma$G affinity column, 47% of rat $^{125}$I-$\gamma$G exchanged with bound and unlabelled $\gamma$G. Finally, with an anti-CEA affinity column, 61% of $^{125}$I-CEA exchanged with bound and unlabelled CEA. By these experiments with 3 different affinity columns, we were unable to repeat the observations of Reisfeld *et al.* (1975) and we must conclude that the criteria described by that group for the demonstration of immunoadsorbent specificity are certainly not universally applicable.

FIG. 1.—Titration of PBS extracts of breast cancer, melanoma and hepatoma against leucocytes from reactive breast-cancer, melanoma and hepatoma patients and from control subjects. The average number of nonadherent cells in triplicate tubes at each dilution of tumour extract is shown. The difference in leucocyte nonadherence to two tumour extracts is expressed as the nonadherence index (NAI).

### RESULTS

Each tumour used for isolation of TSA was tested as a PBS extract in the tube LAI assay against PBL from an appropriately reactive cancer patient and a normal control subject. Fig. 1 shows the number of non-adherent leucocytes from a breast-cancer patient and a control subject when incubated with various concentrations of a PBS extract of either breast cancer or hepatoma. At protein concentrations of 100 to 150 $\mu$g/tube, the leucocytes from the breast-cancer patient showed a significantly greater degree of nonadherence in the presence of the breast-cancer extract than of the hepatoma extract. In contrast, the leucocytes from the control subject showed almost equal degrees of nonadherence in the presence of the tumour extracts at all protein concentrations (Fig. 1). The breast-cancer patient had a positive NAI, whereas the control subject had a negative NAI. Identical results for the leucocytes from patients with malignant melanoma and hepatoma, with their corresponding tumour extracts and appropriate controls, were noted (Fig. 1). Fig. 1 shows that the optimum tumour-directed LAI response (NAI value) is observed at 100 $\mu$g protein/tube, and as previously observed (Flores *et al.*, 1977; Grosser *et al.*, 1976; Grosser and Thomson,

1975, 1976; Marti *et al.*, 1976; Marti and Thomson, 1976) does not have a quantifiable dose-response.

*Chromatographic isolation of papain-solubilized membrane tumour antigens*

In previous studies, the PBS extract of breast cancer was chromatographed on Sepharose 4B and the antigenic activity was shown to be present in the void volume (Grosser and Thomson, 1975). When purified tumour membranes from breast cancer and melanoma were used as antigen in the tube LAI assay, it was shown that the leucocytes from patients with breast cancer and melanoma had LAI reactivity to the tumour membranes of the corresponding histopathological type of tumour (Thomson *et al.*, 1976).

In the present study, when the isolated tumour membranes were digested with papain to yield a water-soluble tumour-antigen preparation, and the papain-soluble membrane material was chromatographed on DEAE-Sephadex A-50 as an initial step to remove highly charged materials, especially DNA, tumour antigenic activity was found in the unbound fraction and not in the bound fraction (data not shown). Up to 60% of the protein was lost in this step (Table XI). The unbound fraction of the papain-soluble breast-tumour material was chromatographed on Sephadex G-150, and Fig. 2 shows the 4 fractions that were pooled for assay by tube LAI. The fractions corresponded roughly to the void volume (Fraction 1), the elution volume of aldolase (Fraction 2), the elution volume of ovalbumin (Fraction 3) and the elution volume of ribonuclease (Fraction 4). In the melanoma preparations, the LAI activity was present principally in Fractions 1 and 2 (Table I). The tumour antigen or LAI activity of papain-soluble membrane from breast cancer was also found principally in Fractions 1 and 2 (Table II). Such activity was occasionally found in Fraction 3 although the activity recovered was generally less than that found in either of Fractions 1 or 2. Fig. 2 shows that Fraction 2 represents material that elutes

| | 1 | 2 | 3 | 4 |
|---|---|---|---|---|
| Total Protein (mg) | 8 | 56 | 52 | 54 |
| Total B2m (ng) | 506 | 3159 | 75,600 | 146,250 |
| Total HLA Activity (μg) | 0.16 | 0.17 | 91.0 | 87.0 |
| LAI Activity | + | ++ | ± | ± |

FIG. 2.—Sephadex G-150 chromatographic pattern of the papain-soluble breast-cancer membranes. The fractions were pooled as shown and the total protein, $\beta_2$m and HLA content in each of the 4 fractions was determined. The elution position of the protein standards is indicated. When the fractions are assayed by tube LAI with leucocytes from reactive breast-cancer patients the usual results are indicated.

in the mol. wt range 70,000–150,000, whereas Fraction 3 includes material eluting in the mol. wt range 30,000–70,000. Material with mol. wt <30,000, found in Fraction 4, infrequently showed LAI activity. Thus, the results indicate that the papain-solubilized tumour-membrane fractions that showed tumour-antigen activity in the standard tube LAI assay eluted principally in the mol. wt range 70,000–150,000 or >150,000 (Tables I and II).

In the tube LAI assay, PBS extracts are used as the standard antigen; hence, in the initial experiments the papain-solubilized material from the cancer-cell membranes were used as the specific antigen, and compared with the PBS cancer extracts as the nonspecific antigens. Tables I and II show that the isolated papain-soluble cancer materials can replace their equivalent PBS tumour extract and retain immunological specific reactivity.

TABLE I.—*Papain-solubilized Melanoma Membranes Chromatographed on Sephadex G-150: Antigenic Activity Assayed by Tube LAI.*

| Donor of melanoma membranes | Donor of leucocytes‡ | NAI* of donor to PBS tumour extract | NAI† of Sephadex G-150 chromatographic fractions 1 | 2 | 3 | 4 |
|---|---|---|---|---|---|---|
| 1 | Mel | 100 | 105 | 97 | 29 | −16 |
| | Con | 3 | 28 | 8 | −3 | −17 |
| 2 | Mel | 52 | 60 | 59 | 19 | −12 |
| | Con | −11 | −15 | −3 | −6 | −12 |
| 3 | Mel | 60 | 56 | 46 | 25 | −11 |
| | Con | 0 | −7 | −4 | −10 | −18 |
| 4 | Mel | 59 | 38 | 90 | −2 | 6 |
| | Con | 16 | −4 | −4 | −2 | −14 |
| 5 | Mel | 47 | 20 | 68 | −14 | 31 |
| | Con | 15 | −17 | 0 | −1 | 19 |
| | Mel | 57 | 14 | 74 | −18 | 35 |
| | Con | 6 | −6 | −3 | −5 | 23 |

*Nonadherence index (NAI) was calculated with PBS extracts of melanoma as specific antigen and of breast cancer as nonspecific antigen.

†NAI was calculated with the papain-soluble melanoma fractions as the specific antigen and the nonspecific antigen was a PBS extract of breast cancer, except in Preparation 4 where the nonspecific antigen was papain-soluble bowel cancer Fractions 1 and 2. The papain-soluble melanoma fractions were tested at ~100 μg/tube. The presence of LAI activity is indicated by an NAI value of 30 or more.

‡Melanoma patient (Mel) or Control subject (Con).

TABLE II.—*Papain-solubilized Breast Cancer Membranes Chromatographed on Sephadex G-150: Antigenic Activity Assayed by Tube LAI.*

| Donor of breast-cancer membranes | Donor of leucocytes‡ | NAI* of donor to PBS tumour extracts | NAI† of Sephadex G-150 chromatographic fractions 1 | 2 | 3 | 4 |
|---|---|---|---|---|---|---|
| 1 | Br. Ca. | 36 | 51 | 79 | 29 | 0 |
| | Con. | 16 | 4 | −27 | 5 | −7 |
| | Br. Ca. | 50 | 69 | 70 | 12 | 13 |
| | Con. | 20 | −5 | −19 | 13 | −8 |
| | Ben Br. | 15 | −14 | −24 | −1 | −7 |
| | Con. | 5 | 26 | −5 | 10 | −11 |
| 2 | Br. Ca. | 114 | 116 | 79 | 38 | −5 |
| | Con. | 19 | −8 | −18 | 10 | −8 |
| | Br. Ca. | 97 | 43 | 48 | 30 | −2 |
| | Con. | −28 | 4 | −7 | 11 | 6 |
| | Ben. Br. | 15 | −18 | −15 | −12 | 5 |
| | Con. | 5 | 1 | −4 | 3 | 12 |
| 3 | Br. Ca. | 57 | 34 | 39 | 14 | 7 |
| | Con. | 18 | −9 | −16 | 2 | 15 |
| | Br. Ca. | 40 | 30 | 33 | 22 | −6 |
| | Con | 5 | 12 | −8 | 14 | −19 |
| | Br. Ca. | 44 | 32 | 50 | 17 | −2 |
| | Con. | 12 | 6 | −10 | −1 | −3 |
| 4 | Br. Ca. | 56 | −2 | 43 | 11 | −11 |
| | Con. | −8 | −26 | −13 | −18 | −20 |
| 5 | Br. Ca. | 113 | 9 | 54 | 44 | 5 |
| | Con. | 18 | 7 | 17 | 3 | −2 |
| 6 | Br. Ca. | 49 | 21 | 87 | 32 | 59 |
| | Con. | 13 | −15 | −24 | 14 | 23 |

*NAI was calculated with PBS extracts of breast cancer as specific antigen and PBS extracts of melanoma and ovarian cancer as nonspecific antigen.

†For Donors 5 and 6, papain-soluble hepatoma membranes and normal liver membranes were the nonspecific control antigens. The third group in Donor 3 was tested with papain-soluble bowel-cancer membranes as the nonspecific control antigens. In the remainder of the preparations, the control nonspecific antigen was a PBS extract of either melanoma or ovarian cancer. The presence of LAI activity is indicated by an NAI value of 30 or more.

‡Breast-cancer patient (Br. Ca.), Control (Con.) or Benign breast disease (Ben. Br.).

Subsequently, the differences in leucocyte reactivity to the specific and nonspecific antigens was compared when both antigenic materials were papain-solubilized extracts of the cancer-cell membranes isolated in an identical fashion (Tables I and II).

The standard tube LAI was highly dependent on protein concentrations. When the protein concentration of the PBS tumour extracts is below 50 μg/tube in the standard tube LAI assay, the number of nonadherent cells decreases (Fig. 1) and the number of nonadherent cells is often too few to consider the counts statistically significant. Similar results occurred when we tried to titrate the isolated papain-soluble tumour antigens to determine the least quantity of material that had antigenic activity; hence, quantitation of antigen activity was not possible with the standard tube LAI assay (Thomson, 1978). To solve this problem, we have recently used the blocking tube LAI assay (Grosser and Thomson, 1976) to detect the presence of TSA activity (Lopez and Thomson, 1977; Thomson, 1978). Table III shows that only the isolated papain-solubilized breast-cancerantigen (Br. Ca. Ag.) blocks reactive leucocytes from breast-cancer patients. Similarly, reactive leucocytes from patients

TABLE III.—*Results of Blocking Assayed by Tube LAI with Papain-Solubilized TSA Chromatographed on Sephadex G-150.*

| Donor of leucocytes | NAI* of leucocyte donor | Donor leucocytes pre-incubated with material from: | Protein concentration of blocking material ($\mu$g) | NAI* after blocking |
|---|---|---|---|---|
| Breast cancer | 54 | Normal breast tissue unbound from DEAE | 400 | 53 |
| | | Fraction 2 | 400 | 59 |
| | | Hepatoma Fractions 2 | 600 | 39 |
| | | 4 | 600 | 55 |
| | | Melanoma Fraction 2 | 200 | 54 |
| | | Breast cancer Fractions 1 | 200 | 62 |
| | | 2 | 200 | −10 |
| | | 3 | 200 | 38 |
| | | 4 | 200 | −22 |
| | | 2 | 100 | 2 |
| | | | 50 | 28 |
| | | | 10 | 61 |
| | | 4 | 100 | 19 |
| | | | 50 | 37 |
| | | | 10 | 66 |
| Malignant melanoma | 71 | Breast cancer Fractions 2 | 600 | 59 |
| | | 4 | 600 | 61 |
| | | Melanoma Fractions 2 | 100 | −6 |
| | | 4 | 100 | 5 |
| Colonic cancer | 58 | Breast cancer Fraction 2 | 200 | 64 |
| | | Colonic cancer Fraction 2 | 100 | −12 |
| | | | 25 | −5 |
| | | | 10 | 51 |

*An NAI value of 30 or more indicates LAI reactivity.

with melanoma and colon cancer were blocked only by the papain-soluble melanoma and colon-cancer membrane material, respectively. The results in Table III show that the amount of material required to block leucocyte reactivity is more easily quantitated. By the blocking assay, TSA activity was measured in 7 different breast-cancer preparations and found to be highest in Fraction 2. Although activity was detected in some preparations in Fractions 1, 3 and 4, the antigen activity was usually less than that in Fraction 2. Similar results were observed in 3 melanoma and 2 colon-cancer preparations.

Because it was not possible to study all fractions, in the present study attention was confined to the material in Fraction 2, since most TSA activity was in this fraction and the TSA present was possibly closer to its native structure.

*SDS-PAGE of tumour-antigen preparations*

Fraction 2 from the Sephadex G-150 column was labelled with $^{125}$I and electrophoresed on 10% SDS gels. The patterns of the materials from Fraction 2 were very similar, regardless of tumour type. Fig. 3 shows the $^{125}$I profile on 10% SDS-gels of papain-soluble breast cancer and melanoma material from Fraction 2 of the Sephadex G-150 column. Two major radioactive peaks with mol. wt of ~12,000 and 40,000, and a relatively minor peak with mol. wt ~25,000 were observed. Similar gel patterns were observed whether or not the gels were run in the presence or absence of the reducing agent mercaptoethanol.

*Guanidine-HCl chromatography of papain-soluble tumour-antigen material*

The $^{125}$I-labelled papain-soluble breast-cancer material from Fraction 2 of the Sephadex G-150 column, composed of material of mol. wt 60,000–150,000, was chromatographed on Sephadex G-200 in 6 M guanidine-HCl and found to consist of smaller subunits (Fig. 4). Moreover, smaller subunits were observed when chromatography was carried out in the presence or absence of dithiothreitol (Fig. 4). Fraction 2 from the Sephadex G-150 column obtained from the papain-solubilized melanoma material, similarly chromatographed, resolved into smaller mol. wt subunits (Fig. 4).

*Isolation of papain-soluble tumour antigen by anti-human-$\beta_2$m affinity chromatography*

Since the analysis of the papain-soluble fractions with tumour-antigen activity indicated that these molecules

FIG. 3.—Electrophoresis on 10% SDS gel of $^{125}$I-labelled breast-cancer material and melanoma material from chromatographic Fraction 2 of Sephadex G-150 (Fig. 2). The mobility of the reduced protein standards is indicated.

FIG. 4.—Upper figure: 6M guanidine-HCl chromatographic profile of $^{125}$I-labelled breast-cancer material from chromatographic Fraction 2 of Sephadex G-150; pattern with $^{125}$I-breast cancer material reduced with dithiothreitol (●) and unreduced (▲).

Lower figure: 6M guanidine-HCl chromatographic profile of reduced $^{125}$I-labelled malignant-melanoma material from chromatographic Fraction 2 of Sephadex G-150. The elution positions of the reduced protein standards are indicated. Each fraction had a volume of 5 ml.

had a macromolecular structure composed of smaller subunits, one of which was of mol. wt ~12,000, this suggested the presence of $\beta_2$m in the preparation, which was in fact demonstrated in the whole preparation by radioimmunoassay for $\beta_2$m. To determine whether the molecule responsible for the observed tumour-antigen activity was linked to $\beta_2$m, the papain-soluble material from Fraction 2 of the Sephadex G-150 column was applied to the horse anti-human-$\beta_2$m affinity column and the bound fractions eluted with 3M KSCN. The unbound fraction contained minimal or no $\beta_2$m, whereas the bound fraction contained high levels of $\beta_2$m, as measured by radioimmunoassay.

The bound and unbound fractions were tested against leucocytes from both specifically LAI reactive patients and control subjects. Table IV shows the LAI response of melanoma patients and control subjects to the unbound and bound melanoma fractions from the anti-$\beta_2$m affinity column. The leucocytes from the melanoma patient showed positive LAI reactivity to the bound melanoma fraction containing $\beta_2$m, contrasting with the negative reaction of the control subject. Moreover, the leucocytes of the reactive melanoma patient showed no LAI reactivity to the

TABLE IV.—*Papain-soluble Melanoma Membranes Isolated by Anti-Human $\beta_2m$ Affinity Chromatography: Antigenic Activity Assayed by Tube LAI**

| | | $\beta_2$m Affinity column fractions | | | | | |
|---|---|---|---|---|---|---|---|
| | | Unbound | | | Bound | | |
| Leucocyte donor | NAI of donor† to PBS tumour extract | Protein‡ μg/tube | $\beta_2$m‡ ng/ml | NAI§ | Protein‡ μg/tube | $\beta_2$m‡ ng/ml | NAI§ |
| Malignant melanoma | 83 | 50 | 0 | 11 | 50 | 18 | 109 |
| Malignant melanoma | 76 | 50 | 0 | 17 | 50 | 18 | 112 |
| Control | −11 | 50 | 0 | −26 | 50 | 18 | −6 |
| Malignant melanoma | 38 | 100 | 0 | −5 | 100 | 24 | 33 |
| Control | −3 | 100 | 0 | −10 | 100 | 24 | −19 |

* Papain-soluble melanoma material was isolated by Sephadex G-150 chromatography and Fraction 2 with LAI activity was applied to the anti-human-$\beta_2$m affinity column.

† Melanoma as specific antigen and with breast cancer as nonspecific antigen.

‡ The isolated unbound and bound fractions had their total protein and $\beta_2$m content measured by the Lowry method and $\beta_2$m radioimmunoassay, respectively.

§ Papain-soluble melanoma material as the specific antigen and a PBS extract of breast cancer as the nonspecific antigen. LAI activity is indicated by an NAI value of 30 or more.

TABLE V.—*Papain-soluble Breast-cancer Membranes Isolated by Anti-Human $\beta_2m$ Affinity Chromatography: Antigenic Activity Assayed by Tube LAI**

| | | $\beta_2$m Affinity column fractions | | | | | |
|---|---|---|---|---|---|---|---|
| | | Unbound | | | Bound | | |
| Leucocyte donor | NAI† of donor to PBS tumour extract | Protein‡ μg/tube | $\beta_2$m‡ ng/ml | NAI§ | Protein‡ μg/tube | $\beta_2$m‡ ng/ml | NAI§ |
| Breast cancer | 57 | 150 | 0 | −28 | 150 | 72 | 80 |
| Control | −18 | 150 | 0 | −27 | 150 | 72 | −29 |
| Breast cancer | 38 | 100 | 0 | 17 | 100 | 48 | 63 |
| Control | −9 | 100 | 0 | −20 | 100 | 48 | −28 |
| Breast cancer | 55 | 30 | 0 | −17 | 30 | 9 | 48 |
| Control | −18 | 30 | 0 | −29 | 30 | 9 | −23 |

* Papain-soluble breast-cancer material was isolated by Sephadex G-150 chromatography and tested for LAI activity. Fraction 2 with proven activity was then fractionated by anti-$\beta_2$m affinity chromatography.

† Breast cancer as the specific antigen and ovarian cancer as the nonspecific antigen.

‡ Total content of protein and $\beta_2$m in the assay tubes was calculated from that present in the whole isolated unbound and bound fractions.

§ Papain-soluble material as specific antigen and the PBS extracts of ovarian cancer as the nonspecific antigen. Presence of LAI activity is indicated by an NAI value of 30 or more.

unbound, $\beta_2$m free, fraction. Identical results were observed when leucocytes from reactive breast-cancer patients and control subjects were tested against unbound and bound papain-soluble breast-cancer fractions from the anti-$\beta_2$m affinity column (Table V). Those fractions that contained $\beta_2$m and specifically reacted in the tube LAI assay also contained HLA antigens, as determined by radioimmunoassay.

Initially, the leucocyte reactivity to the papain-solubilized membrane material from cancers isolated by anti-$\beta_2$m affinity chromatography was compared to a nonspecific PBS tumour extract, and Tables IV and V show that the antigens isolated from the anti-$\beta_2$m affinity columns can replace their equivalent PBS tumour extract and retain specific antigen activity. Next, papain-soluble material from hepatoma membranes was isolated by anti-$\beta_2$m affinity chromatography, and this material, rather than PBS tumour extracts, was used as the control antigen in the tube LAI assay (Table VI). The leucocytes from the reactive melanoma patient showed LAI activity against the bound melanoma fraction from the anti-$\beta_2$m affinity column (Table VI). Likewise, leucocytes of reactive breast patients displayed LAI activity to the bound breast-cancer fraction from the anti-$\beta_2$m affinity column. Leucocytes from the melanoma and breast-cancer patients showed no reactivity to the corresponding unbound fractions. By contrast, leucocytes of the control subjects showed no LAI activity either to the bound or to the unbound fractions.

A reactive hepatoma patient and a control subject were tested against the unbound and bound hepatoma and breast fractions from the anti-$\beta_2$m immunoadsorbent affinity column (Table VII).

TABLE VI.—*Papain-soluble Melanoma Antigen and Breast-cancer Antigen Isolated by Anti-Human-$\beta_2m$ Affinity Chromatography and Assayed by Tube LAI**

| Leucocyte donor | NAI* of donor to PBS tumour extract | Source of papain-† soluble antigen | $\beta_2$m Affinity column fractions: Unbound $\beta_2$m‡ ng/ml | Unbound NAI§ | Bound $\beta_2$m‡ ng/ml | Bound NAI§ |
|---|---|---|---|---|---|---|
| Malignant melanoma | 40 | | | −25 | | 75 |
| | | Melanoma III | <1 | | 20 | |
| | | Hepatoma I | <1 | | 8 | |
| Control | 5 | | | −19 | | 9 |
| Breast cancer | 43 | | | 14 | | 45 |
| | | Breast Cancer II | 0 | | 100 | |
| | | Hepatoma I | <1 | | 8 | |
| Control | −4 | | | 23 | | 11 |

*Melanoma and breast cancer as specific antigen with a melanoma patient and a breast-cancer patient, respectively, and PBS hepatoma extract as nonspecific antigen.

†Papain-soluble tumour materials were chromatographed on Sephadex G-150, and Fraction 2 with specific LAI activity was isolated by anti-$\beta_2$m affinity chromatography.

‡$\beta_2$m measured by radioimmunoassay.

§Melanoma or breast-cancer isolate as specific antigen and the hepatoma isolate as the nonspecific antigen. Each assay tube had 100 μg protein.

Leucocytes from the hepatoma patient showed LAI reactivity to both the bound and unbound hepatoma fractions, whereas leucocytes from the control showed no LAI reactivity. However, Table VIII shows that, in this instance, the affinity column was overloaded with $\beta_2$m-containing material, so that both the unbound and bound hepatoma fractions contained significant quantities of measurable $\beta_2$m. The unbound fraction containing $\beta_2$m was re-applied to the anti-$\beta_2$m immunoadsorbent affinity column and the unbound and bound fractions were retested against the leucocytes from the reactive hepatoma patient and the control subject. After this recycling leucocytes from the hepatoma patient had LAI reactivity only to the bound fraction containing $\beta_2$m and not to the unbound fraction that was now free of $\beta_2$m. Leucocytes from the control subject showed no LAI reactivity.

TABLE VII.—*Papain-soluble Hepatoma Antigen Isolated by Recycling on Anti-Human-$\beta_2m$ Affinity Chromatography and Assayed by Tube LAI.*

| Leucocyte donor | NAI* of donor to PBS tumour extract | Source of Papain-† soluble antigen | $\beta_2$m Affinity column fractions: Unbound $\beta_2$m‡ ng/ml | Unbound NAI§ | Bound $\beta_2$m‡ ng/ml | Bound NAI§ |
|---|---|---|---|---|---|---|
| Hepatoma | 42 | | | 86 | | 92 |
| | | Hepatoma II¶ | 7 | | 308 | |
| | | Breast cancer | 0 | | 150 | |
| Control | 8 | | | 28 | | 15 |
| Hepatoma | 42 | | | 52 | | 33 |
| | | Hepatoma II** | 5 | | 246 | |
| | | Breast cancer | 0 | | 100 | |
| Control | 8 | | | 20 | | 0 |
| After recycling unbound papain-soluble hepatoma material‖ | | | | | | |
| Hepatoma | 42 | | | 22 | | 90 |
| | | Hepatoma II¶ | 0 | | 80 | |
| | | Breast cancer | 0 | | 150 | |
| Control | −7 | | | 15 | | −23 |

*Hepatoma as specific antigen and breast cancer as the nonspecific antigen.

†Papain-soluble tumour materials were chromatographed on Sephadex G-150 and Fraction 2 with LAI activity was isolated by anti-$\beta_2$m affinity chromatography.

‡Measured by radioimmunoassay.

§Soluble hepatoma material as specific antigen and breast-cancer material as nonspecific antigen.

‖The unbound fraction and the bound fraction from the 2nd passage of the hepatoma material through the anti-$\beta_2$m column were tested for LAI activity.

¶125 μg/tube.

**100 μg/tube.

To determine if the papain-soluble cancer antigens bound specifically to the anti-$\beta_2$m immunoadsorbent column, papain-soluble breast-cancer material was applied to immunoadsorbent columns of normal IgG from both humans and rabbits. About 85% of the material applied to the columns remained in the unbound fraction, and ~7% of the material applied was recovered in the bound fraction by elution with 3 M KSCN. The unbound material retained the antigenic activity when

TABLE VIII.—*Results of Application and Recycling of Papain-Soluble Hepatoma Antigen on Anti-Human-$\beta_2m$ Affinity column.*

| | Total Protein (mg) | Total $\beta_2m$ (ng) |
|---|---|---|
| Applied | 59 | 23,000 |
| Unbound | 22 | 1,203 |
| Bound | 13 | 32,000 |
| Reapplication of unbound | | |
| Applied | 19 | 1,054 |
| Unbound | 12 | <56 |
| Bound | 1·2 | 960 |

assayed by tube LAI, whereas the bound fraction had no activity (Table IX). In addition, by $\beta_2m$ radioimmunoassay no $\beta_2m$ was in the bound fraction, whereas the unbound fraction had $\beta_2m$ activity (Table IX).

In the preceding studies (Tables IV, V, VI and VII) the papain-solubilized materials from the membranes of cancer cells isolated by anti-$\beta_2m$ affinity chromatography were shown to behave similarly to the PBS cancer extracts, in inhibiting leucocyte adherence to glass in a way that was immunologically specific. However, because the standard tube LAI assay is highly dependent on protein concentrations, the blocking tube LAI was used to quantitate the purification of TSA activity in different tumour preparations from the anti-$\beta_2m$ column (Table X). The breast- and colon-cancer and melanoma antigens isolated by anti-$\beta_2m$ affinity column were consistently able to block at 1 $\mu$g and, in some preparations, at 0·5 $\mu$g (Table X). By comparison, Table III shows that, before purification by anti-$\beta_2m$ affinity chromatography, 50 and 25 $\mu$g of material from Fraction 2 of the breast and colon, respectively, were required to block LAI reactivity. Table X shows that the blocking was immunologically specific, since the leucocytes of the breast-cancer patient were not blocked by hepatoma or melanoma antigen isolated in the same way from the anti-$\beta_2m$ affinity column. Likewise, leucocytes from the melanoma patient were blocked by melanoma antigen but not by breast- or colon-cancer antigen, and leucocytes from the colon-cancer patient were blocked by the colon-cancer antigen but not by the melanoma antigen isolated from the anti-$\beta_2m$ affinity column. Leucocytes from 5 breast-cancer patients were tested against 4 breast-cancer preparations with identical results. Similarly, leucocytes from 3 different melanoma patients and 2 colon-cancer patients were tested against 2 melanoma and 2 colon-cancer preparations with identical results. Moreover, the reactivity of leucocytes to the specific tumour material that bound to the anti-$\beta_2m$ affinity column and the tumour material which did not bind was highly significant ($P<0{\cdot}001$).

In addition, the binding of the TSA to the anti-$\beta_2m$ affinity column was specific, since the TSA did not bind to control affinity columns (Table X). Passage of the bound material from the anti-$\beta_2m$ column through an anti-NHS column did not remove the blocking activity, indicating that the antigen had bound specifically to

TABLE IX.—*Papain-soluble Breast Cancer Antigen Applied to an Immunoadsorbent Affinity Column of Normal IgG: Antigenic Activity Assayed by Tube LAI§*

| Leucocyte donor | NAI of donor to PBS tumour extract | Source of papain-* soluble antigen | Affinity column fractions: Unbound $\beta_2m$† ng/ml | Unbound NAI§ | Bound $\beta_2m$‡ ng/ml | Bound NAI§ |
|---|---|---|---|---|---|---|
| Breast cancer | 42 | | | 47 | | 2 |
| | | Breast cancer III | 14 | | 0 | |
| | | Liver | 9 | | 0 | |
| Control | −5 | | | −10 | | 7 |
| Control | 7 | | | −13 | | 12 |

*Papain-soluble materials were chromatographed on Sephadex G-150 and Fraction 2 with specific LAI activity was applied to an AH-Sepharose 4B column linked with normal IgG.

†Measured by radioimmunoassay.

‡Breast cancer as specific antigen and liver as the nonspecific antigen.

§IgG was from normal human sera. Similar results were observed when papain-soluble breast-cancer antigen was passed through an affinity column of IgG from normal rabbits.

the anti-$\beta_2$m column (Table X). Similarly, papain-soluble breast material from Sephadex G-150 Fraction 2 was passaged through an affinity column of normal rabbit IgG and the antigen activity was found in the unbound fraction, indicating again that binding to the anti-$\beta_2$m column was specific (Table X).

Also, antisera to non-$\beta_2$m cell-surface proteins were raised in rabbits to papain-soluble breast-cancer and melanoma membrane material that had not bound to the horse anti-$\beta_2$m affinity column. The IgG (unabsorbed) derived from these antisera was linked to AH-Sepharose 4B, along with antiserum to NHS that had been absorbed on papain-soluble liver-cell membranes linked to AH-Sepharose 4B. On this column, which had only recently been prepared, the papain-solubilized TSA of bowel cancer in Sephadex G-150 Fraction 2 material was shown not to bind, indicating that antisera prepared to cell-surface proteins excluding $\beta_2$m and $\beta_2$m-linked proteins does not bind the colon TSA (Table X).

TABLE X.—*Results of Blocking Assayed by the Tube LAI After Passage of Papain-Soluble TSA Through an Anti-Human-$\beta_2$m Affinity Column and Control Columns*

| Donor of leucocytes | NAI of leucocyte donor | Donor leucocytes preincubated with material from: | Protein concentration of blocking material ($\mu$g) | NAI* after blocking |
|---|---|---|---|---|
| Reactive Breast | | *Anti-human-$\beta_2$m affinity fractions of:* | | |
| | | Breast cancer | | |
| cancer A | 38 | unbound | 500 | 55 |
| | | bound | 100 | −4 |
| B | 37 | unbound | 500 | 35 |
| | | bound | 100 | 7 |
| C | 58 | bound | 100 | 0 |
| | | | 10 | −5 |
| | | | 1·0 | 11 |
| | | | 0·5 | 34 |
| D | 50 | bound | 100 | −7 |
| | | | 1 | 8 |
| | | | 0·5 | −7 |
| | | | 0·1 | 38 |
| | | Hepatoma | | |
| | | unbound | 500 | 52 |
| | | bound | 200 | 48 |
| | | Melanoma | | |
| | | unbound | 500 | 59 |
| | | bound | 200 | 43 |
| Malignant melanoma | | | | |
| | | Breast cancer | | |
| A | 38 | unbound | 500 | 49 |
| | | bound | 200 | 60 |
| | | Breast cancer | | |
| B | 111 | bound | 100 | 84 |
| | | Colonic cancer | | |
| | | bound | 100 | 98 |
| | | Melanoma | | |
| | | unbound | 500 | 76 |
| | | bound | 100 | 23 |
| | | | 1·0 | 13 |
| | | | 0·5 | 50 |
| | | | 0·1 | 63 |
| Colonic cancer | 66 | Colonic cancer | | |
| | | unbound | 100 | 59 |
| | | bound | 100 | 13 |
| | | | 1·0 | 11 |
| | | | 0·5 | 42 |
| | | Melanoma | | |
| | | bound | 100 | 52 |
| Breast D cancer | | *Anti-NHS affinity† fractions of: bound from anti-human-$\beta_2$m* | | |
| | | Breast cancer | | |
| | 46 | unbound | 500 | −28 |
| | | bound | 50 | 35 |
| Malignant melanoma C | 37 | unbound | 50 | 34 |
| Breast cancer | | *Normal rabbit IgG affinity fractions of:* | | |
| | | Breast cancer | | |
| | 55 | unbound | 100 | −29 |
| | | bound | 200 | 38 |
| Colonic cancer | | *Anti-non-$\beta_2$m affinity fractions of:* | | |
| | | Colonic cancer fraction 2 | | |
| | 42 | unbound | 50 | −4 |
| | | bound | 50 | 58 |

*An NAI value of 30 or more indicates LAI reactivity.

†The bound papain-soluble breast-cancer material from the anti-human-$\beta_2$m affinity column was passaged through an anti-NHS affinity column and the unbound and bound material was tested for blocking.

TABLE XI.— *Protein Yields in Isolation of Papain-solubilized TSA*

| | | Preparation | | |
|---|---|---|---|---|
| | | Breast cancer | Malignant melanoma | Hepatoma |
| Wet weight of tumour processed (g) | | 460 | 303 | 307 |
| Purified membranes (mg) | | 4747 | 2114 | 2788 |
| Papain-soluble protein (mg) | | 425 | 208 | 147 |
| DEAE chromatography | unbound (mg) | 172 | 111 | 122 |
| Sephadex G-150 chromatography (mg) | Fraction | | | |
| | 1 | 44 | 12 | 15 |
| | 2 | 40 | 25 | 27 |
| | 3 | 59 | 39 | 39 |
| | 4 | 46 | 36 | 13 |
| Affinity chromatography with anti-human-$\beta_2$m (mg) | applied | 31 (10,150)* | 14 (1,378) | 20 (684) |
| | unbound | 18 (0) | 8 (144) | 7 (80) |
| | bound | 7 (7,200) | 5 (1,056) | 3 (258) |

*In parentheses, quantity of $\beta_2$m (ng) in fractions measured by double-antibody radioimmunoassay after a single passage through the anti-human-$\beta_2$m immunoadsorbent column.

The passage of papain-solubilized tumour membrane from Fraction 2 through the horse anti-$\beta_2$m affinity column completely deleted from the material its LAI activity, associated with a proportionate loss of the weight of the starting material. A variable loss of antigenic activity occurred during the brief exposure to the 3M KSCN, due to denaturation. Also, the results in Tables VIII and XI indicate that recovery of the material applied to the anti-human-$\beta_2$m affinity column was not complete. The loss was variable and ranged between 20 and 40%. Typical yields of materials from each step during the isolation of the papain-solubilized TSA are shown in Table XI. The yields were fairly consistent on each occasion.

In fractions with no $\beta_2$m, no LAI activity was found. On the other hand, free $\beta_2$m from urine or normal liver tissue showed no activity in the tube LAI assay. Hence, the antigen that is reactive in the LAI is a molecule that is associated with $\beta_2$m but is not $\beta_2$m itself. $\beta_2$m was present in all fractions from the Sephadex G-150 column, and the quantity increased in the lower mol. wt fractions (Fig. 3). Although Fraction 2 does not have large quantities of $\beta_2$m, the tumour-antigen activity eluted principally in this mol. wt fraction and was associated with $\beta_2$m.

*SDS–PAGE of material isolated by the anti-$\beta_2$m affinity column*

Fig. 5 shows the 10%-SDS-gel electrophoresis profile of $^{125}$I-labelled material from the bound fraction of the anti-$\beta_2$m affinity column. Breast cancer, melanoma and hepatoma material appear to be composed of 2 major subunits with mol. wts of ~12,000 and 40,000. A minor peak was observed at ~25,000. Although the hepatoma material showed a major peak between 40,000 to 35,000, it was diffuse and appeared more heterogeneous with more or less distinct peaks with mol. wts of ~35,000, 40,000 and 50,000. Also, the major peak at ~40,000 from the melanoma and breast material showed heterogeneity. The small irregularities observed in the contour of the peak were seen in the same preparation run on separate occasions, and in different preparations prepared in a similar manner.

To be certain that the materials isolated from the anti-$\beta_2$m affinity column were not substances that had "bled" from the column, and were subsequently labelled with $^{125}$I, antigenic material to be applied to the affinity column was prelabelled and the $^{125}$I profiles of the material applied, unbound and bound, were then analysed by 10% SDS–PAGE. Fig. 6 shows the $^{125}$I profiles. The material bound to the column has a similar profile to that when the material was labelled after elution from the affinity column. This indicates that contamination by "bleeding" from the column did not, in these instances at least, significantly alter the profile on gel electrophoresis.

Next, the material run in the SDS-tube gels was electrophoresed on high-resolution Laemmli polyacrylamide-SDS slab gels (0·75 mm thick with a running gradient of 5 to 20% polyacrylamide) to

FIG. 5.—SDS–PAGE of the $^{125}$I-labelled Papain-solubilized tumour materials (breast, melanoma and hepatoma respectively) that bound and were eluted from the horse anti-human-$\beta_2$m affinity column. The papain-soluble material that bound and was eluted with 3M KSCN from the affinity column was shown to have specific LAI activity. The eluted materials were labelled with $^{125}$I and run reduced on 10% SDS gels. The mobility of the reduced protein standards is indicated.

FIG. 6.—The papain-solubilized malignant-melanoma material from Sephadex G-150 (Fraction 2) was prelabelled with $^{125}$I and applied to the anti-human-$\beta_2$m affinity column. The SDS–PAGE profiles of the applied, unbound and bound materials are shown.

determine the pattern on staining with Coomassie blue. Fig. 7 shows the Coomassie-blue SDS–PAGE profile of papain-soluble breast-cancer material isolated from the horse anti-human-$\beta_2$m immunoadsorbent affinity column. Slot 7 shows the pattern of papain-soluble breast-cancer material bound and eluted from an immunoadsorbent column of *normal* rabbit IgG. Since only small quantities of the papain-soluble material adhered to the control column, it was necessary to concentrate the material in contrast to that of the material bound and eluted from the

FIG. 7.—Coomassie blue stain of isolated papain-soluble breast-cancer material after PAGE on slab gel with a 5–20% gradient of polyacrylamide. Slots 3, 4 and 8 show the protein standards (from top to bottom): phosphorylase (90,000); albumin (68,000); ovalbumin (43,000)); chymotrypsinogen (25,000); and cytochrome C (12,400). Slot 7: papain-soluble breast-cancer material (Fraction 2) bound and eluted from an immunoadsorbent column of *normal* IgG. Slot 1: a preparation of papain-soluble breast-cancer material bound and eluted from an immunoadsorbent horse anti-human-$\beta_2$m. Slot 2: the same preparation as applied to Slot 1 except the material had been passed through an immunoadsorbent column of rabbit anti-human-whole-serum. Slots 5 and 6: another preparation of papain-soluble breast cancer material bound and eluted from an immunoadsorbent column of horse anti-human-$\beta_2$m. The gel was overloaded to detect minor contaminants and slot 6 had twice as much protein as Slot 5. The papain-soluble breast-cancer material shown in Slots 1, 2, 5 and 6 had TSA activity in the tube LAI assay, whereas the material in Slot 7 had no LAI activity.

horse anti-$\beta_2$m immunoadsorbent affinity column (Slots 1, 2, 5, 6). In Slot 7, the most prominent bands are in the mol. wt ranges ~50,000 and 24,000, and probably represent IgG that has bled from the control immunoadsorbent column. Analysis of this material by radioimmunoassays for $\beta_2$m and HLA revealed no $\beta_2$m or xenoantigenic HLA activity; the material also had no LAI activity. By contrast, a papain-soluble breast-cancer preparation bound and eluted from a horse anti-$\beta_2$m affinity column is shown in Slots 5 and 6. Slots 5 and 6 were intentionally overloaded with breast-cancer material isolated from the anti-$\beta_2$m affinity column, to detect minor contaminants. A major band with a mobility in the mol. wt range 40,000–43,000 is seen. This band corresponds to the major radiolabelled peak of ~40,000 mol. wt observed on the 10% SDS gels. However, the band stains far more intensely than would be expected from the height of the radiolabelled peak, and suggests that the material is not readily radiolabelled. By contrast, the stained band of $\beta_2$m at ~12,000 mol. wt is barely visible, although a prominent radioactive peak is observed on SDS gels. Bands at ~25,000 mol. wt are also seen by Coomassie blue stain and they correspond approximately to the area where radiolabelled peaks are seen on SDS gels. A band at ~80,000 mol. wt is shown by staining on the slab gels but was not detected on the radiolabelled gels (Figs. 3, 5 and 6). This could represent a small residual amount of aggregated material that was not reduced, or an entirely separate protein moiety. In Slots 1 and 2, the patterns of yet another preparation of papain-soluble breast-cancer material isolated from the horse anti-$\beta_2$m affinity column are apparent. In this instance, the amount of protein applied was appropriate for the capacity of the gels. A prominent band is present at about 40,000 mol. wt and the band at 11,000–12,000 mol. wt is faint. A protein band at ~30,000 is also prominent. A very faint band is barely visible at ~80,000 mol. wt. In addition, the papain-soluble breast-cancer material run in Slot 2 had been passed through an immunoadsorbent affinity column of rabbit anti-NHS. A comparison of the bands in Slots 1 and 2 reveals no differences.

Autoradiography of the material in Slot 2 revealed intense bands at ~12,000, 26,000, and 40,000 mol. wt, with the bands at 12,000 and 26,000 being stronger than the 40,000 band; other bands were also seen though they were much fainter (Fig. 8). The material electrophoresed in Slot 2, after passage through the rabbit anti-human-normal-serum affinity column, retained its antigen activity in the tube LAI assay (Table X). The breast TSA isolated from the immunoadsorbent column of horse anti-human-$\beta_2$m that gave the patterns seen in Slots 1, 2, 5 and 6 (Fig. 7) had specific LAI activity when assayed by the standard and blocking tube LAI and contained $\beta_2$m and HLA xenoantigenic activity by radioimmunoassay.

The papain-soluble colon-cancer material isolated by anti-$\beta_2$m affinity chromatography when electrophoresed on the high-resolution SDS slab gels and stained with Coomassie blue showed bands at ~12,000, 25,000, 40,000 and 50,000 mol. wt. The band at 50,000 and a portion of the material at 25,000 mol. wt probably represent the H and L chains of IgG that bled from the column during elution with 3M KSCN. A faint band was also observed at ~65,000 mol. wt. The unbound and bound papain-soluble colonic-cancer material from the anti-non-$\beta_2$m affinity column were run on the high-resolution SDS slab gel and stained with Coomassie blue. In this instance, the unbound colon material showed intense bands at ~12,000, 25,000, and 40,000 mol. wt, whereas the pattern of the bound material was entirely different and lacked these 3 prominent bands.

Xenoantiserum raised to the papain-soluble Br.Ca.Ag. and absorbed with normal human serum bound $^{125}$I-$\beta_2$m-Br.Ca.Ag. and $^{125}$I-HLA but not $^{125}$I-$\beta_2$m (Table XII). After the $^{125}$I-HLA or $^{125}$-$\beta_2$m-Br.Ca.Ag. had been initially precipitated

FIG. 8.—Autoradiograph of isolated papain-soluble breast-cancer material shown in Slot 2 of Fig. 7.

with an excess of the xenoantiserum to either HLA or $\beta_2$m-Br.Ca.Ag., no additional material could be subsequently immunoprecipitated with the other xenoantiserum. This indicated that both xenoantiserum recognized similar xenoantigenic determinants on the heavy chain of HLA. Next, $^{125}$I-$\beta_2$m-Br.Ca.Ag. was reacted with xenogeneic anti-human $\beta_2$m, anti-HLA, anti-$\beta_2$m-Br.Ca.Ag. and normal rabbit serum, and then *Staphylococcus aureus* Cowan 1 was used as a bacterial adsorbent, as described by Kessler (1975). The adsorbed immune complexes were released by boiling for 2 min in 2% SDS buffer and 2% mercaptoethanol, and the freed immune complexes were run in SDS gels by the discontinuous method of Laemmli (1970). Similar peaks of radioactivity were observed at ~40,000, 25,000, 12,000 mol. wt with immune complexes produced by the xenoantisera to $\beta_2$m, HLA and $\beta_2$m-Br.Ca.Ag. Normal rabbit serum gave no radiolabelled peaks.

### DISCUSSION

In the present study, the standard and blocking tube LAI assays were used to monitor the isolation of tumour antigen of 4 different types of human cancer. The tumour antigen, solubilized from the tumour cell membranes by papain, was isolated by techniques routinely used for

TABLE XII.—*Results of Binding Studies with Xenoantisera to Cell-surface Antigens*

| | % Antigen precipitated‡ | | | |
|---|---|---|---|---|
| Xenoantisera[1] | $^{125}$I-$\beta_2$m | $^{125}$I-HLA | $^{125}$I-$\beta_2$m-Br.Ca.Ag.[2] | |
| Anti-human-$\beta_2$m | 93 | 24 | 46 | 43 |
| Anti-human-HLA | 4 | 32 | 51 | 48 |
| Anti-$\beta_2$m-Br.Ca.Ag. | 3 | 12 | 28 | 24 |
| Normal rabbit serum | 4 | 4 | 12 | 7 |

*Used at 1/100 dilution.

† Two different isolates of papain-soluble Br.Ca.Ag. from anti-human-$\beta_2$m affinity column.

‡ After incubation of $^{125}$I-labelled antigen and xenoantisera overnight at 4°C, an excess (100 $\mu$l) of goat anti-rabbit-IgG or goat anti-horse IgG was added. Immunes complexes were pelleted by centrifugation at 20,000 $g$ for 20 min, the precipitate was washed once with PBS and the supernatant and pellet were counted in a gamma counter.

purification of human HLA antigens (Peterson, Rask and Lindblom, 1974). Immunochemical studies revealed that the tumour antigen molecules co-isolated with material indistinguishable physicochemically from HLA antigens, which undoubtedly represented the major constituents in the materials isolated. The TSAs bore a distinct similarity to normal human HLA antigens in their linkage to $\beta_2$m (Peterson *et al.*, 1974).

In the present study, when the isolated papain-soluble TSA and reactive leucocytes were from patients with cancers of similar origin and histology, the papain-soluble TSA was able to inhibit the glass adherence of the leucocytes to a greater extent than papain-soluble TSA or PBS tumour extracts from an unrelated tumour. However, the standard tube LAI assay performed in serum-free medium is highly dependent on protein concentration (Grosser and Thomson, 1975; Marti and Thomson, 1976; Flores *et al.*, 1977; Lopez and Thomson, 1977; Thomson, 1978) and titration of the isolated papain-soluble antigens below 50 $\mu$g becomes unreliable because the number of nonadherent cells is too few to be counted with any consistency. Hence, a blocking tube LAI assay (Grosser and Thomson, 1976; Lopez and Thomson, 1977; Thomson, 1978) was used to quantitate the TSA activity in the isolates. The advantages of the blocking tube LAI over the standard tube LAI for the detection of TSA in tumour isolates has recently been discussed (Thomson, 1978). Moreover, in the present study, on each occasion care was taken to show that TSA activity detected by either assay was immunologically specific. Although it appeared highly unlikely that the papain-soluble TSAs would absorb quantitatively in a nonspecific fashion to the immunoadsorbent column, this possibility was excluded, since TSA activity was not removed by immunoadsorbent columns of either IgG derived from antisera to non-$\beta_2$m cell-surface proteins or to NHS or IgG derived from normal human and rabbit serum. Similarly, immunoadsorbent columns used in the isolation of TSA from the serum of patients with metastatic breast cancer were shown not to bind the TSA in a nonspecific manner (Lopez and Thomson, 1977).

After Sephadex G-150 chromatography, the majority of the soluble TSA was present in the fraction that eluted in the mol. wt range 70,000–150,000. TSA activity was present in the excluded fraction; however, this fraction was not extensively studied because of the possibility that it could represent, in part, unsedimented membrane fragments rather than water-soluble TSA. TSA activity was detected in other fractions but usually less frequently and with less activity. Possible explanations for some variability in the elution position of the papain-soluble TSA may be related to differences in papain digestion, the extent of aggregation with storage and/or the degree of autolysis of original tumour samples. Hence, in the present study attention was focused on the further isolation and characterization of the material containing TSA activity that eluted in the mol. wt range 70,000–150,000.

Analysis of the material with TSA activity in the 70,000–150,000 mol. wt range by SDS–PAGE and 6M guanidine-HCl column chromatography indicated that the material was composed of smaller subunits. The presence of a prominent band on SDS gels at ~12,000 mol. wt suggested that the material contained $\beta_2$m, and this was confirmed by $\beta_2$m radioimmunoassay of the whole material. Hence, affinity chromatography with horse anti-human-$\beta_2$m was undertaken to determine whether the TSAs might be associated with $\beta_2$m. The results showed that the papain-solubilized TSAs from tumours of 4 different origins and histology bound specifically to the anti-$\beta_2$m affinity column. Analysis of the bound material from the 4 different tumours by SDS–PAGE consistently showed major peaks at about 12,000, 25,000 and 40,000 mol. wt. Thus, papain-soluble material from the cell membranes of breast and bowel cancers, melanoma and hepatoma isolated by anti-$\beta_2$m affinity chromatography have similar molecular weights and subunit structures. It is not known, however, whether the chain that carries the TSA epitope is on the 25,000 or 40,000 chains; moreover, it is possible that the chain carrying the TSA epitope is not visualized by the SDS gels.

The studies of Nakamuro, Tanigaki and Pressman (1977) have suggested that $\beta_2$m is exclusively combined with the HLA large component, and no other membrane components are involved in binding $\beta_2$m. Likewise, Robb *et al.* (1976) claim that detergent-solubilized HLA antigen is rapidly purified with very little contamination by affinity chromatography with rabbit anti-human-$\beta_2$m. The fact, therefore, that the papain-soluble human TSAs from breast and bowel cancer, melanoma and hepatoma bound specifically to the ant-$\beta_2$m immunoadsorbent suggests that the TSA determinant is possibly an integral part of the HLA molecule or alternatively, is physically associated with the HLA molcule. Moreover, breast TSA isolated from serum and urine also co-isolates with HLA antigens and appears to share the same xenoantigenic determinants as present on the heavy chains of HLA antigens (Lopez and Thomson, 1977). Obviously, not all the molecules that are noncovalently linked with $\beta_2$m are TSAs since even if the TSA is eventually proved to be a modified HLA antigen, it is unlikely that all 4 of the individual allogeneic molecules would be altered (Blank and Lilly, 1977).

Not surprisingly, rabbits immunized with papain-soluble breast-cancer material isolated from the anti-$\beta_2$m affinity column and containing TSA activity did not result in the rabbit producing a reagent that recognized the TSA determinant. Antisera prepared in rabbits, even to purified HLA antigens, are not likely to produce reagents of great value in revealing HLA allospecificity (Sanderson, 1977). The rabbit can be expected to differ from man at several places other than the epitope on the HLA chain, so allospecific globulins would comprise a minor portion of the total antibody formed against the whole alloantigen chain with its attendant $\beta_2$m (Sanderson, 1977). Similarly, the protein chain in humans that carries the TSA epitope, whatever the origin of the protein, might be expected to differ from rabbit at several sites other than the TSA epitope.

In addition, transplantation antigens solubilized from normal and neoplastic tissues with limited papain digestion demonstrate non-H-2 activity as well as H-2 activity, as indicated by skin-graft rejection (Graff and Nathenson, 1971). Recent biochemical evidence suggests that the antigenic products of both T/t and H-2 are structurally similar, and $t^{12}$ has been reported to be associated with a $\beta_2$m-like moiety in the membrane (Artzt and Bennett, 1975; Michailson *et al.*, 1977). Similar results have been reported for the TL antigen (Artzt and Bennett, 1975; Michailson *et al.*, 1977; Ostberg *et al.*, 1975) which is closely linked to H-2D and apparently has a reciprocal interaction with it in the plasma membrane. In addition, in the region between H-2D and T/a, genes specify for a cell surface molecule Qa-2 which is similar to H-2D in molecular weight and association with $\beta_2$m (Michailson *et al.*, 1977). It is suggested that a family of molecules related by size, subunit structure, genetic linkage, membrane location and antigenicity may exist in this area of the chromosome, and may have arisen from a common ancestral gene (Michailson *et al.*, 1977). Thus, in humans, similar non-HLA antigens may exist and structurally resemble antigens of the HLA complex; and these non-HLA antigens could possibly be involved in the expression of cell-surface TSAs.

Evidence has accumulated, however, to support the concept that HLA antigens represent the markers of self-recognition. The existence of such a mechanism provides a biological basis for the evolution of strong transplantation antigen systems, a known physiological role for which has been lacking. Doherty, Blanden and

Zinkernagel (1976) have proposed that the T cell recognizes an "altered self" antigen formed by an interaction between H-2 coded structures at the D and K regions of major histocompatibility complex (MHC) and the "inducing" antigen. To date, the data from studies with haptenated cells (Shearer, Rehn and Garbarino, 1975), non-H-2 alloantigens (Bevan, 1975) including the sex-linked H-Y antigens (Gordon, Simpson and Samelson, 1975), viral infected cells (Zinkernagel and Doherty, 1974) and a haptenated H-2-deficient tumour line (Forman and Vitetta, 1975) support the altered-self interaction antigen (Bevan, 1975) adaptor-antigen complex (Schrader and Edelman, 1976) hypothesis. Nevertheless, the mechanism by which antigen associates with MHC-coded gene products to form the new altered self or altered antigen is not yet clear. Moreover, the existence of altered self antigens has not been shown biochemically.

Human TSAs could possibly be of 3 different origins:

1. The TSA could be a modified MHC-coded structure;

2. The TSA could be spatially linked with the HLA antigens but on independently coded molecules with the HLA molecules serving as adaptors that combine with antigens on the cell surface to form hybrid antigens containing elements of self (HLA) and non-self (TSA) (Ohno, 1977; Schrader, Cunningham and Edelman, 1975). In this context, however, a preliminary examination of our breast-cancer tumour isolates for associated murine mammary-tumour viral antigens have given negative results;*

3. TSA could be a molecule that is unrelated to HLA antigens and is expressed separately on the cell surface, but may share some xenoantigenic determinants and/or structural features with the HLA antigens.

The data from our studies are insufficient to determine which, if any, of the above possibilities is correct. Nevertheless, the results of the present study provide a unified approach to the isolation of human TSA from cancers arising in different organs.

* Our anti-$\beta_2$m-Br.Ca.Ag. serum does not precipitate any of the MMTV polypeptides, and the radiolabelled $\beta_2$m-Br.Ca.Ag. was not precipitable with antisera to MMTV polypeptides. The cold $\beta_2$m-Br.Ca.Ag. did not inhibit radiolabelled virus in RIA; if putative MMTV antigen was present, the concentration was $< 1$ ng/50 $\mu$l. These studies were kindly performed for us by Dr R. D. Cardiff, Dept. of Pathology, University of California, School of Medicine, Davis, California.

The expert assistance of Drs J. Marti and M. Flores in certain of these studies is appreciated. The authors thank Dr S. O. Freedman, Director of the Division of Clinical Immunology and Allergy, where this work was performed, for his support and for his review of the manuscript. The authors are grateful to Dr W. P. Duguid, Pathologist-in-Chief for the pathology specimens; and to Ms Mary Naughton for the preparation of this manuscript. This work was supported by the Medical Research Council of Canada, Ottawa, Canada, and the Cancer Research Society Inc. of Montreal.

### REFERENCES

ARTZT, K. & BENNETT, D. (1975) Analogies Between Embryonic (T/t) Antigens and Adult Major Histocompatibility (H-2) Antigens. *Nature*, **256**, 545.

BALDWIN, R. W. & BARKER, C. R. (1967) Demonstration of Tumour-Specific Humoral Antibody Against Aminoazo Dye-Induced Rat Hepatoma. *Br. J. Cancer*, **21**, 793.

BALDWIN, R. W. & GLAVES, D. (1972) Solubilization of Tumour-Specific Antigen from Plasma Membrane of an Aminoazo Dye-Induced Rat Hepatoma. *Clin. exp. Immun.*, **11**, 51.

BEVAN, M. J. (1975) The Major Histocompatibility Complex Determines Susceptibility to Cytotoxic T Cells Directed Against Minor Histocompatibility Antigens. *J. exp. Med.*, **142**, 1349.

BLANK, K. J. & LILLY, F. (1977) Evidence for an H-2/Viral Protein Complex on the Cell Surface as the Basis for the H-2 Restriction of Cytotoxicity. *Nature*, **269**, 808.

BOWEN, J. G. & BALDWIN, R. W. (1975) Tumour-Specific Antigen Related to Rat Histocompatibility Antigens. *Nature*, **258**, 75.

COMOGLIO, P. M., BESTINI, M. & FORNI, G. (1975) Evidence for a Membrane Carrier Molecule Common to Embryonal and Tumour-specific Antigenic Determinants Expressed by a Mouse Transplantable Tumour. *Immunology*, **29**, 353.

CRESSWELL, P. & AYRES, J. L. (1976) HLA Antigens: Rabbit Anti Sera Reacting with All A Series or All B Series Specificities. *Eur. J. Immun.*, **6**, 82.

DOHERTY, P. C., BLANDEN, R. V. & ZINKERNAGEL, R. M. (1976) Specificity of Virus Immune Effector T Cells for H-2K or H-2D Compatible Interactions: Implications for H-Antigen Diversity. *Transplant. Rev.*, **29**, 89.

FLORES, M., MARTI, J. H., GROSSER, N., MACFARLANE, J. K. & THOMSON, D. M. P. (1977) An

Overview: Antitumour Immunity in Breast Cancer Assayed by Tube Leukocyte Adherence Inhibition. *Cancer*, **39**, 494.

FORMAN, J. & VITETTA, E. S. (1975) Absence of H-2 Antigens Capable of Reacting with Cytotoxic T Cells on a Teratoma Line Expressing a T/t Locus Antigen. *Proc. natn. Acad. Sci.*, **72**, 3661.

FUJISAWA, T., WALDMAN, S. R. & YONEMATO, R. H. (1977) Leukocyte Adherence Inhibition by Soluble Tumor Antigens in Breast Cancer Patients. *Cancer*, **39**, 506.

GOODING, L. R. & EDIDIN, M. (1974) Cell Surface Antigens of a Mouse Testicular Teratoma. Identification of an Antigen Physically Associated with H-2 Antigens on Tumour Cells. *J. exp. Med.*, **140**, 61.

GORDON, R. D., SIMPSON, E. & SAMELSON, L. E. (1975) *In Vitro* Cell-mediated Immune Responses to the Male Specific (H-Y) Antigen in Mice. *J. exp. Med.*, **142**, 1108.

GRAFF, R. J. & NATHENSON, S. G. (1971) Immunogenic Properties of Papain Solubilized Alloantigen. *Transplant. Proc.*, **3**, 249.

GREENWOOD, F. C., HUNTER, W. M. & GLOVER, J. S. (1963) The Preparation of $^{131}$I-labelled Human Growth Hormone of High Specific Radioactivity. *Biochem. J.*, **89**, 114.

GROSSER, N., MARTI, J. H., PROCTOR, J. W. & THOMSON, D. M. P. (1976) Tube Leukocyte Adherence Inhibition Assay for the Detection of Anti-Tumour Immunity. I. Monocyte is the Reactive Cell. *Int. J. Cancer*, **18**, 39.

GROSSER, N. & THOMSON, D. M. P. (1975) Cell-mediated Anti-tumor Immunity in Breast Cancer Patients Evaluated by Antigen-Induced Leucocyte Adherence Inhibition in Test Tubes. *Cancer Res.*, **35**, 2571.

GROSSER, N. & THOMSON, D. M. P. (1976) Tube Leukocyte (Monocyte) Adherence Inhibition Assay for the Detection of Anti-tumor Immunity. III. "Blockade" of Monocyte Reactivity by Excess Free Antigen and Immune Complexes in Advanced Cancer Patients. *Int. J. Cancer*, **18**, 58.

HALLIDAY, W. J., MALUISH, A., LITTLE, J. H. & DAVIS, N. C. (1975) Leukocyte Adherence Inhibition and Specific Immunoreactivity in Malignant Melanoma. *Int. J. Cancer*, **16**, 645.

HALLIDAY, W. J. & MILLER, S. (1972) Leukocyte Adherence Inhibition: A Simple Test for Cell-mediated Tumour Immunity and Serum Blocking Factors. *Int. J. Cancer*, **9**, 477

HOLAN, V., HASEK, M., BUBENIK, J. & CHUTNO, J. (1974) Antigen-mediated Macrophage Adherence Inhibition. *Cell. Immun.*, **13**, 107.

HOLMES, E. C., KAHAN, B. D. & MORTON, D. L. (1970) Tumour-specific Transplantation Antigens from Methylcholanthrene-Induced Guinea Pig Sarcomas. *Cancer*, **25**, 373.

INVERNIZZI, G. & PARMIANI, G. (1975) Tumour-associated Transplantation Antigens of Chemically Induced Sarcomata Cross-reacting with Allogeneic Histocompatibility Antigens. *Nature*, **254**, 713.

KESSLER, S. W. (1975) Rapid Isolation of Antigens from Cells with a Staphylococcal Protein A-Antibody Absorbent: Parameters of the Interaction of Antibody–Antigen Complexes with Protein A. *J. Immun.*, **115**, 1617.

LAEMMLI, U. K. (1970) Cleavage of Structural Proteins During the Assembly of the Head of Bacteriophage T4. *Nature*, **227**, 680.

LEVESON, S. H., HOWELL, J. H., HOLYOKE, E. D. & GOLDROSEN, M. H. (1977) Leukocyte Adherence Inhibition: An Automated Microassay Demonstrating Specific Antigen Recognition and Blocking Activity in Two Murine Tumor Systems. *J. Immunologic Meth.*, **17**, 153.

LOPEZ, M. J. & THOMSON, D. M. P. (1977) Isolation of Breast Cancer Tumour Antigen from Serum and Urine. *Int. J. Cancer.*, **20**, 834.

LOWRY, O. H., ROSEBROUGH, N. J., FARR, A. L. & RANDALL, R. J. (1951) Protein Measurements with the Folin Phenol Reagent. *J. biol. Chem.*, **193**, 265.

MARTI, J. H., GROSSER, N. & THOMSON, D. M. P. (1976) Tube Leukocyte Adherence Inhibition Assay for the Detection of Anti-tumour Immunity. II. Monocyte Reacts with Tumour Antigen Via Cytophilic Anti-tumour Antibody. *Int. J. Cancer*, **18**, 48.

MARTI, J. H. & THOMSON, D. M. P. (1976) Anti-tumour Immunity in Malignant Melanoma Assay by Tube Leucocyte Adherence Inhibition. *Br. J. Cancer*, **34**, 116.

MICHAILSON, J., FLAHERTY, L., VITETTA, E. & POULIK, M. D. (1977) Molecular Similarities Between the Qa-2 Alloantigen and Other Gene Products of the 17th Chromosome of the Mouse. *J. exp. Med.*, **145**, 1066.

NAKAMURO, K., TANIGAKI, N. & PRESSMAN, D. (1977) Common Antigenic Structures of HLA Antigens VII. Selective Combination Binding of $\beta_2$ microglobulin with HLA Large Component in Cultured Human Cell Lines. *Immunology*, **32**, 139.

OHNO, S. (1977) The Original Function of MHC Antigens as the General Plasma Membrane Anchorage. Site of Organogenesis-directing Proteins. *Immunological Rev.*, **33**, 59.

OLD, L. J. & BOYSE, E. A. (1964) Immunology of Experimental Tumours. *Ann. Rev. Med.*, **15**, 167.

OSTBERG, L., RASK, L., WIGZELL, H. & PETERSON, P. A. (1975) Thymus Leukaemia Antigen Contains $\beta_2$ Microglobulin. *Nature*, **253**, 735.

PETERSON, P. A., RASK, L. & LINDBLOM, J. B. (1974) Highly Purified Papain-solubilized HLA Antigens Contain $\beta_2$ Microglobulin. *Proc. natn. Acad. Sci. U.S.A.*, **71**, 35.

POWELL, A. E., SLOSS, A. M., SMITH, R. N., MAKLEY, J. T. & HUBAY, C. E. (1975) Specific Responsiveness of Leukocytes to Soluble Extracts of Human Tumours. *Int. J. Cancer*, **16**, 905.

REISFELD, R. A., SEVIER, E. D., PELLEGRINO, M. A., FERRONE, S. & POULIK, M. D. (1975) Association of HL-A Antigens and $\beta_2$ Microglobulin at the Cellular and Molecular Level. *Immunogenetics*, **2**, 183.

ROBB, R. J., STROMINGER, J. L. & MANN, D. L. (1976) Rapid Purification of Detergent-solubilized HLA Antigen by Affinity Chromatography Employing Anti-$\beta_2$-microglobulin Serum. *J. biol. Chem.*, **251**, 5427.

RUTHERFORD, J. C., WALTERS, B. A. J., CAVAYE, G. & HALLIDAY, W. J. (1977) A Modified Leukocyte Adherence Inhibition Test in the Laboratory Investigation of Gastrointestinal Cancer. *Int. J. Cancer*, **19**, 43.

SANDERSON, A. R. (1977) HLA "help" for Human $\beta_2$-Microglobulin Across Species Barriers. *Nature*, **269**, 414.

SCHRADER, J. W., CUNNINGHAM, B. A. & EDELMAN, G. M. (1975) Functional Interactions of Viral and

Histocompatibility Antigens at Tumor Cell Surfaces. *Proc. natn. Acad. Sci.*, **12**, 5066.

SCHRADER, J. W. & EDELMAN, G. M. (1976) Participation of the H-2 Antigens of Tumor Cells in their Lysis by Syngenetic T. cells. *J. exp. Med.*, **143**, 601.

SHEARER, G. M., REHN, T. G. & GARBARINO, C. A. (1975) Cell Mediated Lympholysis of Trimitrophenyl-modified Autologous Lymphocytes. Effector Cell Specificity to Modified Cell Surface Components by the H-2K and H-2D Serological Regions of the Murine Major Histocompatibility Complex. *J. exp. Med.*, **141**, 1348.

SMITH, M., GOLD, P., FREEDMAN, S. O. & SHUSTER, J. (1975) Studies of the Linkage Relationship of Beta-$_2$-microglobulin in Man-mouse Somatic Cell Hybrids. *Ann. Human Genet.*, **39**, 21.

THOMSON, D. M. P. (1978) Antigens of Breast Cancer. In *Methods in Cancer Research.* Ed. H. Busch. London, New York: Academic Press (in press).

THOMSON, D. M. P. & ALEXANDER, P. (1973) A Cross-reacting Embryonic Antigen in the Membrane of Rat Sarcoma Cells which is Immunogenic in the Syngeneic Host. *Br. J. Cancer*, **27**, 35.

THOMSON, D. M. P., GOLD, P., FREEDMAN, S. O. & SHUSTER, J. (1976) The Isolation and Characterization of Tumour-specific Antigens of Rodent and Human Tumours. *Cancer Res.*, **36**, 3518.

THOMSON, D. M. P., SELLENS, V., ECCLES, S. & ALEXANDER, P. (1973) Radioimmunoassay of Tumour Specific Transplantation Antigen of a Chemically Induced Rat Sarcoma: Circulating Soluble Tumour Antigen in Tumour Bearers. *Br. J. Cancer*, **28**, 377.

WEBER, K. & OSBORN, M. (1969) The Reliability of Molecular Weight Determinations by Dodecyl Sulfate polyacrylamide Gel Electrophoresis. *J. biol. Chem.*, **244**, 4406.

ZINKERNAGEL, R. M. & DOHERTY, P. C. (1974) Immunological Surveillance Against Altered Self Components by Sensitized T Lymphocytes in Lymphocytic Choriomeningitis. *Nature*, **251**, 547.

ZOLLER, M. & MATZKU, S. (1976) Antigen and Antibody Purification by Immunoadsorption: Elimination of Non-biospecifically Bound Proteins. *J. immunol. Meth.*, **11**, 287.